# Identification of Novel Diarylpyrimidines as Potent HIV-1 Non-Nucleoside Reverse Transcriptase Inhibitors by Exploring the Primer Grip Region

**DOI:** 10.3390/ph15111438

**Published:** 2022-11-19

**Authors:** Tao Zhang, Zhongxia Zhou, Fabao Zhao, Zihao Sang, Erik De Clercq, Christophe Pannecouque, Dongwei Kang, Peng Zhan, Xinyong Liu

**Affiliations:** 1 Key Laboratory of Chemical Biology (Ministry of Education), Department of Medicinal Chemistry, School of Pharmaceutical Sciences, Cheeloo College of Medicine, Shandong University, 44 West Culture Road, Jinan 250012, China; 2 Department of Pharmacy, Shandong Cancer Hospital and Institute, Shandong First Medical University and Shandong Academy of Medical Sciences, Jinan 250117, China; 3 Laboratory of Virology and Chemotherapy, Rega Institute for Medical Research, K.U. Leuven, Herestraat 49 Postbus 1043 (09.A097), B-3000 Leuven, Belgium; 4 China-Belgium Collaborative Research Center for Innovative Antiviral Drugs of Shandong Province, 44 West Culture Road, Jinan 250012, China

**Keywords:** HIV-1, reverse transcriptase, NNRTIs, diarylpyrimidines

## Abstract

HIV-1 reverse transcriptase (RT) plays a crucial role in the viral replication cycle, and RT inhibitors can represent a promising pathway in treating AIDS. To explore the primer grip region of HIV-1 RT, using -CH_2_O- as a linker, substituted benzene or pyridine rings were introduced into the left wing of diarylpyrimidines (DAPYs). A total of **17** compounds with new structures were synthesized. It showed that all compounds exhibited anti-HIV-1 (wild-type) activity values ranging from 7.6–199.0 nM. Among them, **TF2** (EC_50_ = 7.6 nM) showed the most potent activity, which was better than that of NVP (EC_50_ = 122.6 nM). Notably, compared with RPV (CC_50_ = 3.98 μM)**, TF2** (CC_50_ > 279,329.6 nM) showed low cytotoxicity. For HIV-1 mutant strains K103N and E138K, most compounds showed effective activities. Especially for K103N, **TF2** (EC_50_ = 28.1 nM), **TF12** (EC_50_ = 34.7 nM) and **TF13** (EC_50_ = 28.0 nM) exhibited outstanding activity, being superior to that of NVP (EC_50_ = 7495.1 nM) and EFV (EC_50_ = 95.1 nM). Additionally, **TF2** also showed the most potent activity against E138K (EC_50_ = 44.0 nM) and Y181C mutant strains (EC_50_ = 139.3 nM). In addition, all the compounds showed strong enzyme inhibition (IC_50_ = 0.036–0.483 μM), which demonstrated that their target was HIV-1 RT. Moreover, molecular dynamics simulation studies were implemented to predict the binding mode of **TF2** in the binding pocket of wild-type and K103N HIV-1 RT.

## 1. Introduction

**Figure 1:**
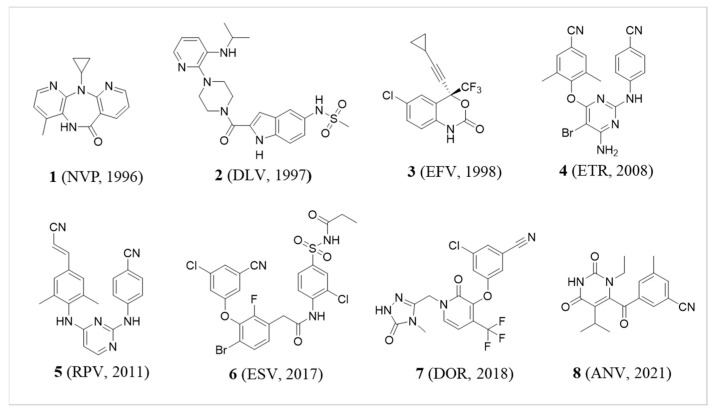
US FDA, Russian MoH and NMPA-approved NNRTI chemical structures.

Human immunodeficiency virus type 1 (HIV-1), one of the viruses that pose a serious threat to human health worldwide, is the main pathogen of acquired immunodeficiency syndrome (AIDS) [1,2,3]. UNAIDS 2022 data indicated that 38.4 million people were living with HIV, 1.5 million people were newly infected with HIV, and 650,000 people have died from AIDS in 2021 [4]. Currently, the clinical treatment for AIDS often uses highly active antiretroviral therapy (HAART), which is a combination of two, three, or more drugs, typically including reverse transcriptase inhibitors (RTIs) [5,6]. Because of the high specificity, potent antiviral properties, and favorable pharmacokinetics, nonnucleoside reverse transcriptase inhibitors (NNRTIs) have proven to be an important part of HAART regimens. The NNRTIs are non-competitive inhibitors that act by allosteric inhibition of DNA polymerization [7,8,9]. There have been six NNRTIs approved by the U.S. Food and Drug Administration (FDA) [10,11], including nevirapine (**1**, NVP), delavirdine (**2**, DLV), efavirenz (**3**, EFV), etravirine (**4**, ETR), rilpivirine (**5**, RPV) and doravirine (**6**, DOR). In addition, elsulfavirine (**7**, ESV) was approved by the Russian Ministry of Health (MoH) in 2017 [12], and ainuovirine (**8**, ANV) was approved by National Medical Products Administration (NMPA) in 2021 for AIDS treatment (Figure 1) [13]. 

Among them, ETR and RPV belong to diarylpyrimidines NNRTIs, exhibiting effective activity against the resistant mutations caused by the first-generation NNRTIs, such as Y181C, P236L and K103N [10,14,15,16]. In spite of their higher genetic barrier, several single, double or triple mutants have emerged with resistance towards these drugs, such as K101E, E138K, Y181V, Y181C + V179F and V179F + Y181C + F227C [17,18,19,20]. Therefore, it is still urgent to develop novel NNRTIs with an improved anti-resistance profile.

**Figure 2:**
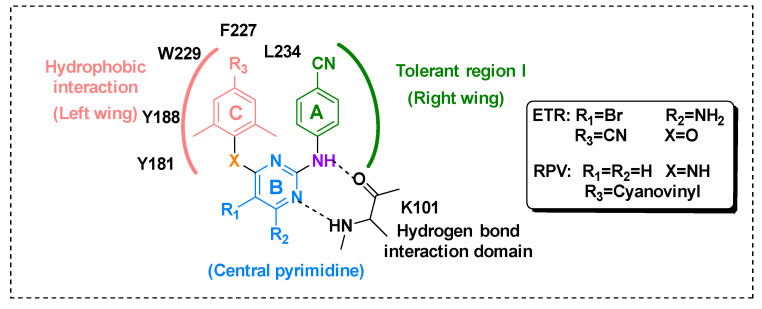
Illustration of the pharmacophore model of diarylpyrimidines.

The rapid development of HIV-1 RT structural biology provides new information for the rational design of potential anti-HIV-1 agents. The RT is a heterodimer consisting of two subunits, p66 (560 residues) and p51 (440 residues), The NNRTI-binding pocket (NNIBP) is located between the *β*6 (residues 142–146), *β*9 (residues 227–229), *β*10 (residues 232–234) sheets (floor) and the *β*12 (residues 326–333), *β*13 (residues 336–344), *β*14 (residues 347–355) sheets (roof) [21,22,23,24]. Diarylpyrimidines tend to bind to HIV-1 RT in a “U” mode (also named “horseshoe mode”), and they can bind to the different NNRTI-binding pockets caused by various resistant RT mutations through torsional flexibility (wiggling) and repositioning flexibility (jiggling) to retain potency against mutant HIV-1 viruses [25,26]. The studies of X-ray crystallography and molecular modeling revealed that diarylpyrimidines exhibited a three-dimensional pharmacophore model in an NNRTI-binding pocket (Figure 2): an aminobenzonitrile moiety (A-ring, right wing), a central pyrimidine ring (B-ring) and the NH linker which form two hydrogen bonds with the backbone of residue K101, and a 2,4,6-trisubstituted phenoxy moiety (C-ring, left wing), and the left wing is situated in the hydrophobic channel which is shaped by amino acid residues Y181, Y188, W229, F227 and L234 [27,28,29]. 

**Figure 3:**
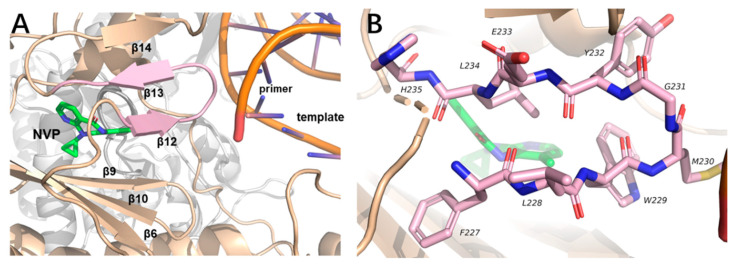
(**A**) NNIBP of HIV-1 RT is shown with nevirapine (green) and primer grip (pink). (**B**) Amino acid composition of primer grip.

The primer grip is a highly conserved structural motif consisting of the β12-β13 hairpin (F227-H235) (Figure 3); it is responsible for locating the 3′-OH end of the primer strand at the polymerase catalytic site, making it important in the catalytic activity of RT [30,31]. The primer grip contains three amino acid residues in the NNRTI-binding pocket: F227, W229 and L234, and some mutations of F227 and L234 have been observed: F227C/L/I/V, L234/I. However, the mutation of the key W229 has never been observed. As for the other amino acid residues in the primer grip region, except for M230 (M230I/L), no other drug-resistance mutations have been reported [32]. Targeting highly conserved amino acid residues is of great significance for the discovery of new compounds with improved drug resistance profiles [33], which inspired us to target the conserved residue W229 in the NNRTI-binding pocket for rational drug design and further explore the chemical space of the primer grip region.

**Figure 4:**
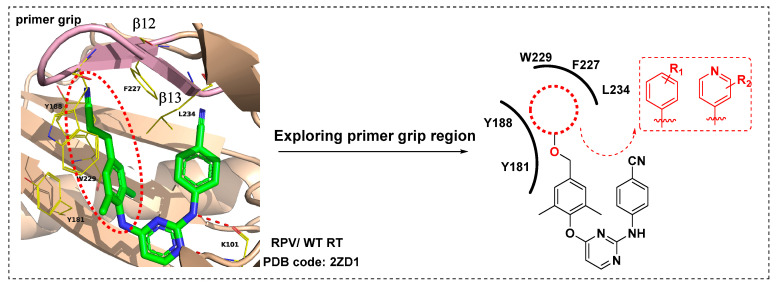
Design strategy of target compounds.

On the basis of the above analysis, with the privileged aminobenzonitrile moiety (right wing) and central pyrimidine ring (B-ring) unchanged, substituted benzene rings or pyridine rings were introduced to the left wing of diarylpyrimidines by a CH_2_O linker, expecting the newly introduced aromatic rings could develop stronger π-π interaction with the conserved residue (W229) and explore primer grip region to enhance the drug resistance profile (Figure 4). Here, we described the synthesis of these novel diarylpyrimidines as well as their anti-HIV-1 activity, and then we further discussed the preliminary structure-activity relationship (SAR) in detail and selected representative molecules for molecular dynamics simulation studies.

## 2. Result and Discussion

### 2.1. Chemistry

**Scheme 1:**
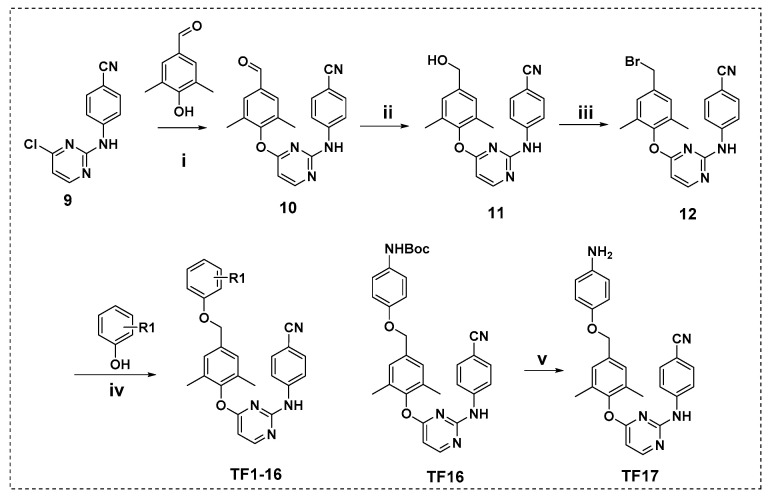
Reagents and conditions: (i) 4-hydroxy-3,5-dimethylbenzaldehyde, K_2_CO_3_, DMF, 100 °C, 10 h; (ii) NaBH_4_, MeOH, 0 °C, 0.5 h, r.t., 4 h; (iii) PBr_3_, CH_2_Cl_2_, 0 °C, 2 h, r.t., 2 h; a (iv) KOH, KI, MeCN, 80 °C, 4 h; (v) CF_3_COOH, CH_2_Cl_2_, r.t., 3 h.

As indicated in Scheme 1, using **9** as the starting material, which we reported earlier [34,35,36], it was subjected to nucleophilic substitution reaction with 3,5-dimethyl-4-hydroxybenzaldehyde under the alkaline condition of potassium carbonate to obtain intermediate **10**. Reduction of **10** with NaBH_4_ gave intermediate **11**, which was brominated with PBr_3_ to give key intermediate **12**. Finally, **12** was reacted with different substituted phenols and thiophenols to obtain the target compounds **TF1-TF16** via nucleophilic substitution reactions. **TF17** was obtained by removing the Boc protecting group of **TF16**.

### 2.2. Biological Evaluation

These 17 newly synthesized compounds were tested in MT4 cells for their antiviral activity using the MTT method [37]. The five marketed drugs approved by the U.S. Food and Drug Administration: Zidovudine (AZT), NVP, EFV, ETR and RPV, were used as positive drugs. EC_50_, CC_50_ and SI were used to express the biological evaluation results, which were summarized in Table 1, Table 2, Table 3 and Table 4.

According to the results in Table 1, all compounds (EC_50_ = 7.6 ~ 199.0 nM) had potent inhibitory activity against WT HIV-1. **TF2** (EC_50_ = 7.6 nM), **TF4** (EC_50_ = 7.8 nM) and **TF12** (EC_50_ = 7.8 nM) exhibited the best anti-HIV-1 activity, being comparable to that of the second-generation drug ETR (EC_50_ = 3.0 nM) and much superior to those of AZT (EC_50_ = 27.3 nM) and NVP (EC_50_ = 122.6 nM). Moreover, at a concentration of 279,329.6 nM, **TF2** exhibited no cytotoxicity, which contributes to its higher SI values (SI > 36,610.9) toward HIV-1 IIIB. None of the compounds had inhibitory activity against HIV-2, which demonstrated that these compounds belong to typical HIV-1 inhibitors. 

The preliminary SAR was summarized as follows. Inhibitory activity differed depending on the types and positions of substituents on the benzene ring introduced to the left wing by a CH_2_O linker. For the compounds containing *para*-substituted benzene ring, the order of potency was as follows: -CN (**TF2**, EC_50_ = 7.6 nM) > -OCH_3_ (**TF5**, EC_50_ = 21.7 nM) > -NO_2_ (**TF15**, EC_50_ = 23.1 nM) > -NH_2_ (**TF17**, EC_50_ = 32.5 nM) > -CH_2_OH (**TF8**, EC_50_ = 42.8 nM) > -NHBoc (**TF16**, EC_50_ = 45.3 nM). For the compounds containing *meta*-substituted benzene ring, the order of potency was as follows: -CN (**TF3**, EC_50_ = 24.5 nM) > -CH_2_OH (**TF9**, EC_50_ = 30.5 nM) > -OCH_3_ (**TF6**, EC_50_ = 35.3 nM). For the compounds containing *ortho*-substituted benzene ring, the order of potency was as follows: -CN (**TF4**, EC_50_ = 7.8 nM) > -OCH_3_ (**TF7**, EC_50_ = 25.5 nM) > -CH_2_OH (**TF10**, EC_50_ = 32.2 nM). Among them, **TF2** and **TF4** showed the best antiviral activity. It preliminarily showed that compared to **TF1** (-H, EC_50_ = 17.1 nM), the introduction of *para*- and *ortho* -CN substituted benzene rings were beneficial to the improvement of antiviral potency.

Furthermore, we found that the substitution of different positions on the introduced benzene rings had a certain effect on cytotoxicity. Compared with compounds containing *ortho*- or *meta*-substituted benzene rings, **TF1** (-H, CC_50_ > 295,865.0 nM), **TF2** (*para*-CN, CC_50_ > 279,329.6 nM), **TF5** (*para*-OCH_3_, CC_50_ = 253,718.1 nM), and **TF15** (*para*-NO_2_, CC_50_ = 215,001.1 nM) showed low cytotoxicity than that of RPV (CC_50_ = 3.89 *µ*M). This suggested that the introduction of the benzene ring and *para*-substituted benzene ring may help reduce cytotoxicity. However, all three compounds containing hydroxymethyl substituted benzene rings, **TF8** (CC_50_ = 2917.9 nM), **TF9** (CC_50_ = 3458.6 nM), **TF10** (CC_50_ = 4804.6 nM), showed a sharp increase in cytotoxicity compared to **TF1**, which suggested that the introduction of hydroxymethyl benzene ring may be detrimental to the improvement of the toxicity of compounds.

In addition, 3-pyridine-containing and substituted 3-pyridine-containing compounds (**TF11-TF14**) were also synthesized. Compared to **TF11** (EC_50_ = 199.0 nM) with an unsubstituted pyridine ring, the activity of **TF12**~**TF14** showed different degrees of increase, among which **TF12** (EC_50_ = 7.8 nM) was the most active compound.

As shown in Table 2, compounds **TF1-17** were further evaluated for their activity against drug-resistant strains of NNRTIs in MT-4 cells (L100I, K103N, Y181C, Y188L, E138K, F227L/V106A and RES056).

For single mutant K103N, all compounds (EC_50_ = 28.0 ~ 444.1 nM) displayed potent inhibitory activity. Notably, **TF2** (EC_50_ = 28.1 nM), **TF12** (EC_50_ = 34.7 nM) and **TF13** (EC_50_ = 28.0 nM) exhibited outstanding activity, which was better than that of NVP (EC_50_ = 7495.1 nM) and EFV (EC_50_ = 95.1 nM). As for the effect of the types and positions of substituents on the introduced benzene ring on the activity, compared with **TF1** (-H, EC_50_ = 50.7 nM), the activity of **TF2** (*para*-CN, EC_50_ = 28.1 nM) was nearly doubled. Moreover, **TF3** (*meta*-CN, EC_50_ = 48.4 nM), **TF5** (*para*-OCH_3_, EC_50_ = 44.5 nM), and **TF15** (*para*-NO_2_, EC_50_ = 46.3 nM) showed comparable activity with **TF1**; it preliminarily showed that the *para*-CN was also the privileged substituent. Regarding the resistance fold (Table 3), compared with EFV (RF = 47.5), the activity of all compounds against the K103N mutant strain was not significantly reduced compared to the wild strain (RF = 1.47 ~ 9.74).

As for single mutant E138K, all compounds showed sub-micromolar inhibitory activity with the exception of **TF11**. Among them, **TF2** (EC_50_ = 44.0 nM) was the most active compound. **TF12** (EC_50_ = 46.2 nM), **TF13** (EC_50_ = 57.7 nM), **TF14** (EC_50_ = 67.4 nM) and **TF15** (EC_50_ = 50.0 nM) also showed promising activities, which were significantly more potent than NVP (EC_50_ = 149.4 nM). Similarly, the compounds with *para*-substituted electron-withdrawing groups on the benzene ring (**TF2** (*para*-CN), **TF15** (*para*-NO_2_) were more active. 

In the case of single mutant strains L100I and Y181C, most of the compounds showed sub-micromolar to micromolar inhibitory activity, **TF2** (EC_50 (L100I)_ = 117.0 nM, EC_50 (Y181C)_ = 139.3 nM) and **TF15** (EC_50 (L100I)_ = 105.2 nM, EC_50 (Y181C)_ = 195.9 nM) had the best activity, which was better than that of NVP (EC_50 (L100I)_ = 1856.7 nM, EC_50 (Y181C)_ = 10113.7 nM). For single mutant Y188L and double mutant F227L + V106A, some compounds were inactive, and most compounds showed sub-micromolar to micromolar inhibitory activity, among which **TF14** (EC_50 (Y188L)_ = 773.6 nM) and **TF12** (EC_50 (F227L + V106A)_ = 742.4 nM) had the best activity, respectively. For the double mutant RES056, unfortunately, only a few compounds showed micromolar inhibitory activity; TF13 (EC_50_ = 1182.9 nM) and **TF14** (EC_50_ = 1192.8 nM) had the best inhibitory activity.

To verify the target of synthesized compounds (**TF1**-**TF17**), they were further tested for enzyme inhibitory activity by the ELISA method [38], and the results are shown in Table 4. All compounds (IC_50_ = 0.038 ~ 0.438 μM) exhibited potent inhibitory activity against WT HIV-1 RT, being superior to that of NVP (IC_50_ = 0.568 μM). The three most potent compounds against WT HIV-1 mutant, **TF2** (IC_50_ = 0.055 μM), **TF4** (IC_50_ = 0.050 μM) and **TF12** (IC_50_ = 0.038 μM), displayed higher enzyme inhibitory activity. The weaker inhibitors of the WT HIV-1 strain showed reduced inhibitory activity, such as **TF11** and **TF16** (IC_50_ = 0.483 and 0.160 μM, respectively). It is noteworthy that the antiviral activity of some compounds was inconsistent with their enzyme-inhibitory potency to some extent. This discrepancy may be caused by the variations in the HIV-1 RT-substrate binding affinities and polymerase processivity on different nucleic acid templates [39]. Nonetheless, these novel synthesized compounds functioned as traditional NNRTIs. 

### 2.3. Molecular Dynamics Simulation Studies

In order to predict the binding modes of these novel synthesized diarylpyrimidines in the NNRTI-binding pocket and initially explain the anti-resistance profiles to the K103N strain of **TF2**, MD simulations were performed in detail using the software Schrödinger [40]. The co-crystal structures of HIV-1 WT RT in complex with RPV (PDB code:2ZD1) [22] and HIV-1 K103N RT in complex with RPV (PDB code:3MEG) [27] were chosen as templates for docking studies.

**Figure 5:**
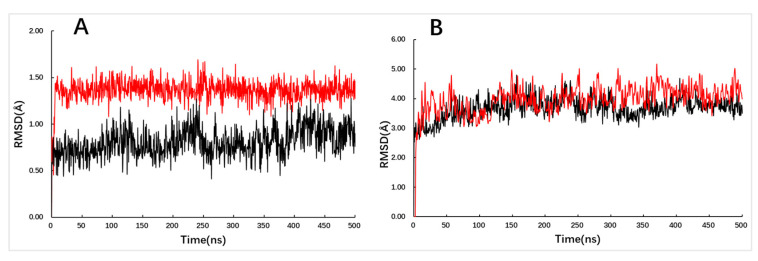
Analysis of MD trajectories for ligand **TF2** in complex with HIV-1WT RT (black) and K103N (red) RT. (**A**) RMSD profiles for the ligand **TF2**, (**B**) RMSD profiles for the protein backbone.

The binding modes of **TF2** to the allosteric pocket in WT and K103N HIV-1 RT were investigated by running a 500 ns MD simulation. Figure 5 shows that the root-mean-square deviation (RMSD) from the initial structure of **TF2** was computed throughout the MD simulation for all systems. Figure 5A illustrates the RMSD of ligand **TF2**; the plot showed that the inhibitor in the WT and K103N mutant strains had deviated from the starting structure. Figure 5B illustrates the RMSD of the complex of RT and ligand **TF2**, which formed different conformational during the MD time. As a result of the narrow range of RMSD values, these conformational ensembles were structurally similar.

**Figure 6:**
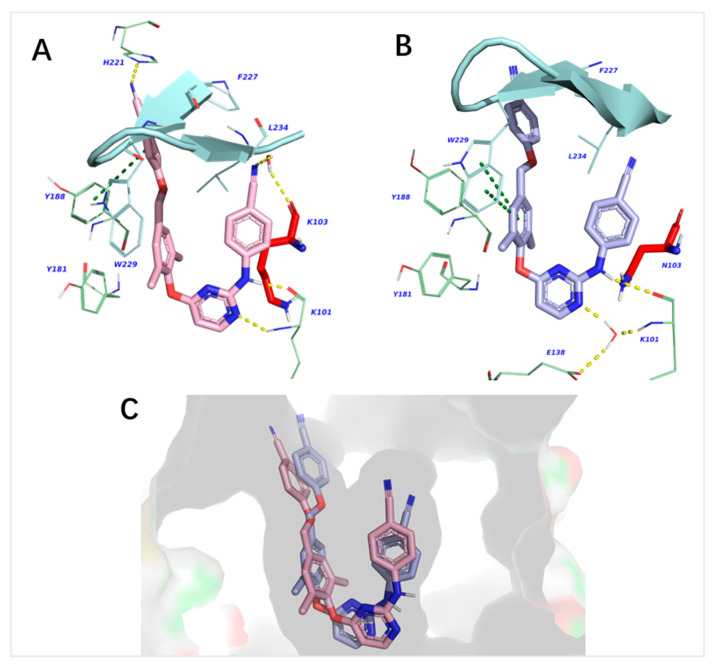
Final complexes of (**A**)WT RT-**TF2**, (**B**) K103N RT—**TF2** obtained from MD simulations. Selected residues are highlighted as pale green lines (carbon atoms in green), the ligand is shown as light pink (carbon atoms) sticks (WT) and light blue (carbon atoms) sticks (K103N), and the primer grip region is colored in pale cyan. (**C**) Superposition of MD structure of the WT RT-**TF2** and K103N RT—**TF2**.

Compound **TF2** bound to WT and K103N RTs in similar binding modes, the binding modes of **TF2** within the NNRTI-binding pocket resembled other diarylpyrimidines as the typical conformation (U shape), as noted in the snapshots collected at the end of the MD trajectories (Figure 6). However, there were some differences in the orientation of some groups and the distance between them and several remarkable features and well-known interactions were delineated here. 

In the WT RT protein (Figure 6A), the right benzonitrile moiety was situated in the tolerant region I, where the cyano group could form a water-mediated hydrogen bond with K103. Stable hydrogen-bonding interactions were formed between the 2-aminopyrimidine ring of **TF2** and the backbone (both NH and C = O units) of K101. The left 2,4,6-trisubstituted moiety remained stable in a hydrophobic sub-pocket formed by Y181, Y188, and W229. Moreover, the newly introduced p-cyanobenzonitrile group formed π-π stacking interaction with Y188 and hydrogen-bonding interaction with an extra amino acid residue H221. In the K103N RT protein (Figure 6B), compared to the binding mode in WT RT, the overall structure of **TF2** was shifted, causing it to move away from K101 and affecting hydrogen-bonding interactions. A hydrogen bond was formed between the pyrimidine-bound NH of **TF2** and the carbonyl oxygen of K101, but the *N* atom on the pyrimidine ring formed water bridge-mediated hydrogen bonds with K101 and E138. Besides, the left 2,4,6-trisubstituted moiety was also positioned in the hydrophobic sub-pocket, and the phenyl group formed π-π stacking interaction with the indole ring of W229. Similarly, the p-cyanophenyl moiety extended out of the hydrophobic channel, but no additional interactions with surrounding amino acids were found.

MMGBSA calculations [41] were used to determine the relative binding strengths of the compound **TF2** to the WT and K103N HIV-1 RTs. These estimations are shown in Table 5. Accordingly, the binding affinity (Δ*G_bind_*) of **TF2** to WT RT was higher than that to K103N RT, supporting the experimental EC_50_ values. Two important interactions observed in the complex of **TF2** and WT were the hydrogen-bonding interaction with an extra residue H221 and a water-mediated hydrogen bond with K103. The H bond (Δ*G_Hbond_*) contributed more to **TF2** binding to WT RT comparing K103N RT, which was consistent with the MD studies.

Overall, a comparison with WT RT revealed that K103N mutation would destabilize the binding mode of **TF2** by partially disrupting the protein-ligand hydrophobic and hydrogen bond interactions, which could also contribute to the reduced activity against this strain. Moreover, the newly introduced *p*-cyanophenyl moiety reached the primer grip region, but no interaction with the key amino acid here was observed. It pointed to the outside of the *β*12, which may not be an ideal location to interact with amino acids in the primer grip region. This also inspired us to pay attention to the selection of linkers to make it more oriented to explore the primer grip region.

## 3. Materials and Methods

### 3.1. Synthesis of Compounds

On a column filled with Silica Gel GF254 for thin-layer chromatography (TLC) and spots were observed using UV radiation at 254 and 365 nm wavelengths. The melting points (mp) of compounds were determined using a micro melting point meter. Flash column chromatography was carried out on a column filled with Silica Gel 60 (200–300 mesh). ^1^H NMR and ^13^C NMR spectra were acquired on a Bruker AV-400 spectrometer with tetramethylsilane as the internal standard and DMSO-*d*_6_ as solvent. The relevant mass spectrometry data were measured by a Standard G1313A Autosampler instrument. Reagents used in this work required no further purification and were bought from commercial sources.

4-((4-(4-Formyl-2,6-dimethylphenoxy)pyrimidin-2-yl)amino)benzonitrile (**10**)

**9** (3.00 g, 13.04 mmol), 4-hydroxy-3,5-dimethylbenzaldehyde (2.35 g, 15.65 mmol) and K_2_CO_3_ (3.60 g, 26.08 mmol) were mixed at 5 mL DMF. The reaction mixture was stirred at 100 °C for 10 h. After the reaction was finished (monitored by TLC), water (40 mL) was added, and the precipitate was filtered to obtain **10**, brown solid, yield: 61%. ^1^H NMR (400 MHz, DMSO-*d*_6_) δ 10.13 (s, 1H, -CHO), 10.03 (s, 1H, NH), 8.50 (d, *J* = 5.6 Hz, 1H, C6-Pyrimidine-H), 7.80 (s, 2H, Benzene-H), 7.56 (d, *J* = 8.6 Hz, 2H, Benzene-H), 7.44 (d, *J* = 8.7 Hz, 2H, Benzene-H), 6.71 (d, *J* = 5.6 Hz, 1H, C5-Pyrimidine-H), 2.17 (s, 6H, 2×CH_3_). ESI-MS: *m/z* calcd for C_20_H_16_N_4_O_2_ 345.13 [M + H]^+^, found 345.4 [M + H]^+^.

4-((4-(4-(Hydroxymethyl)-2,6-dimethylphenoxy)pyrimidin-2-yl)amino) benzonitrile (11)

**10** (1.00 g, 2.91 mmol) was dissolved in 30 mL MeOH, then NaBH_4_ (0.10 g, 2.91 mmol) was added slowly in portions at 0 °C. The reaction was stirred for 0.5 h at 0 °C and for a further 4 h at room temperature. A saturated brine solution (30 mL) was added, and the mixture was extracted with ethyl acetate (3 × 20 mL) after the reaction solution was concentrated under reduced pressure. Then, the organic layer was dried with anhydrous sodium sulfate. Flash column chromatography was used to separate **11** from the mixture with ethyl acetate and petroleum ether, white solid, yield: 45%. ^1^H NMR (400 MHz, DMSO-*d*_6_) δ 10.11 (s, 1H, NH), 8.44 (d, *J* = 5.6 Hz, 1H, C6-Pyrimidine-H), 7.59 (d, *J* = 8.7 Hz, 2H, Benzene-H), 7.50 (d, *J* = 8.8 Hz, 2H, Benzene-H), 7.15 (s, 2H, Benzene-H), 6.60 (d, *J* = 5.6 Hz, 1H, C5-Pyrimidine-H), 5.25 (t, *J* = 5.6 Hz, 1H, OH), 4.52 (d, *J* = 5.6 Hz, 2H, CH_2_), 2.07 (s, 6H, 2×CH_3_). ESI-MS: *m/z* calcd for C_20_H_18_N_4_O_2_ 347.14 [M + H]^+^, found 347.3 [M + H]^+^.

4-((4-(4-(Bromomethyl)-2,6-dimethylphenoxy)pyrimidin-2-yl)amino)benzonitrile (**12**)

**11** (1.00g, 2.88 mmol) was dissolved in 20 mL dichloromethane, then PBr_3_ (0.33 mL, 3.46 mmol) was dissolved in 1.5 mL dichloromethane and added dropwise to the reaction solution at 0 °C. The reaction mixture was stirred for 2 h at 0 °C and for a further 2 h at room temperature until TLC detection of the reaction was complete. A saturated brine solution (30 mL) was added, and the mixture was extracted with ethyl acetate (3 × 20 mL) after the reaction solution was concentrated under reduced pressure. Then, the organic layer was dried with anhydrous sodium sulfate. Flash column chromatography was used to separate **12** from the mixture with ethyl acetate and petroleum ether, white solid, yield: 51%. ^1^H NMR (400 MHz, DMSO*-d*_6_) δ 10.14 (s, 1H, NH), 8.47 (d, *J* = 5.6 Hz, 1H, C6-Pyrimidine-H), 7.56 (d, *J* = 8.6 Hz, 2H, Benzene-H), 7.52 (d, *J* = 8.2 Hz, 2H, Benzene-H), 7.32 (s, 2H, Benzene-H), 6.66 (d, *J* = 5.6 Hz, 1H, C5-Pyrimidine-H), 4.79 (d, *J* = 18.3 Hz, 2H, CH_2_), 2.07 (s, 6H, 2×CH_3_). ESI-MS: *m/z* calcd for C_20_H_17_BrN_4_O 409.06 [M + H]^+^, found 409.4 [M + H]^+^.

#### 3.1.1. General Synthesis Procedure for the Preparation of **TF1**-**TF16**

Intermediate **12** (0.25 mmol), different substituted thiophenols (0.30 mmol), KOH (0.30 mmol) and KI (0.30 mmol) were mixed in 10 mL MeOH. Stirred at 80 °C for 4 h, and the reaction was detected by TLC. Dichloromethane (3 × 20 mL) was used to extract the reaction solution after adding saturated sodium chloride solution (30 mL); then, the organic layer was dried with anhydrous sodium sulfate. Using flash column chromatography, the target compounds were separated from ethyl acetate/petroleum ether.

4-((4-(2,6-dimethyl-4-(phenoxymethyl)phenoxy)pyrimidin-2-yl)amino) benzonitrile (**TF1**)

White solid, yield: 50%, mp: 179–182 °C . ^1^H NMR (400 MHz, DMSO-*d*_6_) δ 10.17 (s, 1H, NH), 8.46 (d, *J* = 5.6 Hz, 1H, C6-Pyrimidine-H), 7.57 (d, *J* = 8.5 Hz, 2H, Benzene-H), 7.49 (d, *J* = 8.6 Hz, 2H, Benzene-H), 7.33 (d, *J* = 9.8 Hz, 4H, Benzene-H), 7.09 (d, *J* = 8.0 Hz, 2H, Benzene-H), 6.97 (t, *J* = 7.3 Hz, 1H, Benzene-H), 6.65 (d, *J* = 5.6 Hz, 1H, C5-Pyrimidine-H), 5.08 (s, 2H, CH_2_), 2.09 (s, 6H, 2×CH_3_). ^13^C NMR (100 MHz, DMSO-*d*_6_) δ 168.97, 160.76, 159.68, 158.95, 149.60, 145.07, 134.90, 133.11, 130.90, 130.01, 129.05, 121.24, 119.90, 118.57, 115.13, 102.90, 99.33, 69.39, 16.55. ESI-MS: *m/z* calcd for C_26_H_22_N_4_O_2_ 423.17 [M + H]^+^, found 423.17 [M + H]^+^.

4-((4-(4-((4-cyanophenoxy)methyl)-2,6-dimethylphenoxy)pyrimidin-2-yl)amino) benzonitrile (**TF2**)

White solid, yield: 62%, mp: 202–205 °C . ^1^H NMR (400 MHz, DMSO-*d*_6_) δ 10.09 (s, 1H, NH), 8.40 (d, *J* = 5.6 Hz, 1H, C6-Pyrimidine-H), 7.76–7.71 (m, 2H, Benzene-H), 7.50 (d, *J* = 8.6 Hz, 2H, Benzene-H), 7.41 (d, *J* = 8.6 Hz, 2H, Benzene-H), 7.27 (s, 2H, Benzene-H), 7.19 (d, *J* = 8.7 Hz, 2H, Benzene-H), 6.58 (d, *J* = 5.6 Hz, 1H, C5-Pyrimidine-H), 5.13 (s, 2H, CH_2_), 2.02 (s, 6H, 2×CH_3_). 13C NMR (100 MHz, DMSO-*d_6_*) δ 168.93, 162.37, 160.80, 159.67, 149.85, 145.07, 134.71, 133.94, 133.09, 131.07, 129.29, 119.91, 119.55, 118.58, 116.27, 103.57, 102.89, 99.33, 70.05, 16.54. ESI-MS: *m/z* calcd for C_27_H_21_N_5_O_2_ 448.17 [M + H]^+^, found 448.38 [M + H]^+^.

3-((4-((2-((4-cyanophenyl)amino)pyrimidin-4-yl)oxy)-3,5-dimethylbenzyl)oxy) benzonitrile (**TF3**)

White solid, yield: 54%, mp: 199–202 °C . ^1^H NMR (400 MHz, DMSO-*d*_6_) δ 10.17 (s, 1H, NH), 8.47 (d, J = 5.7 Hz, 1H, C6-Pyrimidine-H), 7.60–7.43 (m, 9H, Benzene-H), 7.34 (s, 2H, Benzene-H), 6.66 (d, J = 5.6 Hz, 1H, C5-Pyrimidine-H), 5.17 (s, 2H, CH_2_), 2.10 (s, 6H, 2×CH_3_). ^13^C NMR (100 MHz, DMSO-*d*_6_) δ 168.94, 160.79, 159.67, 159.08, 149.82, 145.07, 134.10, 133.09, 131.36, 131.04, 129.31, 125.26, 121.00, 119.92, 119.10, 118.59, 118.03, 112.83, 102.90, 99.33, 70.11, 16.54. ESI-MS: *m/z* 448.38 calcd for C_27_H_21_N_5_O_2_ 446.17 [M-H]^−^, found 446.44 [M-H]^−^.

2-((4-((2-((4-cyanophenyl)amino)pyrimidin-4-yl)oxy)-3,5-dimethylbenzyl)oxy) benzonitrile (**TF4**)

White solid, yield: 61%, mp: 198–201 °C . ^1^H NMR (400 MHz, DMSO-*d*_6_) δ 10.15 (s, 1H, NH), 8.47 (d, *J* = 5.6 Hz, 1H, C6-Pyrimidine-H), 7.77 (dd, *J* = 7.7, 1.7 Hz, 1H, Benzene-H), 7.70 (td, *J* = 8.0, 7.5, 1.7 Hz, 1H, Benzene-H), 7.61 (d, *J* = 8.5 Hz, 2H, Benzene-H), 7.48 (d, *J* = 8.6 Hz, 2H, Benzene-H), 7.43 (d, *J* = 8.6 Hz, 1H, Benzene-H), 7.35 (s, 2H, Benzene-H), 7.14 (t, *J* = 7.6 Hz, 1H, Benzene-H), 6.65 (d, *J* = 5.6 Hz, 1H, C5-Pyrimidine-H), 5.30 (s, 2H, CH_2_), 2.11 (s, 6H, 2×CH_3_). ^13^C NMR (100 MHz, DMSO-*d*_6_) δ 168.93, 160.72, 160.47, 159.71, 149.73, 145.01, 135.52, 134.30, 133.91, 133.17, 131.07, 128.77, 121.84, 119.86, 118.57, 116.88, 114.01, 103.03, 101.35, 99.42, 70.34, 16.64. ESI-MS: *m/z* calcd for C_27_H_21_N_5_O_2_ 448.17 [M + H]^+^, found 448.30 [M + H]^+^.

4-((4-(4-((4-methoxyphenoxy)methyl)-2,6-dimethylphenoxy)pyrimidin-2-yl) amino)benzonitrile (**TF5**)

White solid, yield: 63%, mp: 188–191 °C . ^1^H NMR (400 MHz, DMSO-*d*_6_) δ 10.16 (s, 1H, NH), 8.46 (d, *J* = 5.6 Hz, 1H, C6-Pyrimidine-H), 7.58 (d, *J* = 8.5 Hz, 2H, Benzene-H), 7.51 (d, *J* = 8.6 Hz, 2H, Benzene-H), 7.30 (s, 2H, Benzene-H), 7.06–7.00 (m, 2H, Benzene-H), 6.92–6.85 (m, 2H, Benzene-H), 6.65 (d, *J* = 5.6 Hz, 1H, C5-Pyrimidine-H), 5.02 (s, 2H, CH_2_), 3.71 (s, 3H, CH_3_), 2.09 (s, 6H, 2×CH_3_). ^13^C NMR (100 MHz, DMSO-*d*_6_) δ 168.98, 160.76, 159.68, 153.97, 153.01, 149.52, 145.08, 135.15, 133.12, 130.85, 128.95, 119.91, 118.60, 116.03, 115.10, 102.90, 99.33, 70.00, 55.82, 16.55. ESI-MS: *m/z* calcd for C_27_H_24_N_4_O_3_ 453.18 [M + H]^+^, found 453.20 [M + H]^+^.

4-((4-(4-((3-methoxyphenoxy)methyl)-2,6-dimethylphenoxy)pyrimidin-2-yl) amino)benzonitrile (**TF6**)

White solid, yield: 63%, mp: 149–152 °C . ^1^H NMR (400 MHz, DMSO-*d*_6_) δ 10.17 (s, 1H, NH), 8.47 (d, *J* = 5.6 Hz, 1H, C6-Pyrimidine-H), 7.59 (d, *J* = 8.5 Hz, 2H, Benzene-H), 7.50 (d, *J* = 8.6 Hz, 2H, Benzene-H), 7.32 (s, 2H, Benzene-H), 7.22 (t, *J* = 8.2 Hz, 1H, Benzene-H), 6.71–6.62 (m, 3H, Benzene-H), 6.56 (dd, *J* = 8.2, 2.3 Hz, 1H, C5-Pyrimidine-H), 5.08 (s, 2H, CH_2_), 3.76 (s, 3H, CH_3_), 2.10 (s, 6H, 2×CH_3_). ^13^C NMR (100 MHz, DMSO-*d*_6_) δ 168.97, 161.04, 160.75, 160.23, 159.69, 149.59, 145.07, 134.85, 133.12, 130.89, 130.45, 129.02, 119.90, 118.60, 107.34, 107.06, 102.92, 101.37, 99.33, 69.56, 55.55, 16.55. ESI-MS: *m/z* calcd for C_27_H_24_N_4_O_3_ 453.18 [M + H]^+^, found 453.25 [M + H]^+^.

4-((4-(4-((2-methoxyphenoxy)methyl)-2,6-dimethylphenoxy)pyrimidin-2-yl) amino)benzonitrile (**TF7**)

White solid, yield: 58%, mp: 208–211 °C . ^1^H NMR (400 MHz, DMSO-*d*_6_) δ 10.15 (s, 1H, NH), 8.47 (d, *J* = 5.7 Hz, 1H, C6-Pyrimidine-H), 7.63 (d, *J* = 8.3 Hz, 2H, Benzene-H), 7.52 (d, *J* = 8.5 Hz, 2H, Benzene-H), 7.31 (s, 2H, Benzene-H), 7.12 (d, *J* = 7.4 Hz, 1H, Benzene-H), 7.01 (d, *J* = 7.5 Hz, 1H, Benzene-H), 6.92 (p, *J* = 7.4 Hz, 2H, Benzene-H), 6.65 (d, *J* = 5.7 Hz, 1H, C5-Pyrimidine-H), 5.06 (s, 2H, CH_2_), 3.78 (s, 3H, CH_3_), 2.10 (s, 6H, 2×CH_3_). ^13^C NMR (100 MHz, DMSO-*d*_6_) δ 168.99, 160.65, 159.72, 149.72, 149.50, 148.45, 145.05, 135.02, 133.19, 130.81, 129.05, 121.71, 121.08, 119.89, 118.66, 114.20, 112.62, 102.99, 99.47, 70.27, 55.95, 16.58.ESI-MS: *m/z* calcd for C_27_H_24_N_4_O_3_ 453.18 [M + H]^+^, found 453.32 [M + H]^+^. 

4-((4-(4-((4-(hydroxymethyl)phenoxy)methyl)-2,6-dimethylphenoxy)pyrimidin-2-yl)amino)benzonitrile (**TF8**)

White solid, yield: 50%, mp: 209–212 °C . ^1^H NMR (400 MHz, DMSO-*d*_6_) δ 10.16 (s, 1H, NH), 8.46 (d, *J* = 5.6 Hz, 1H, C6-Pyrimidine-H), 7.57 (d, *J* = 8.6 Hz, 2H, Benzene-H), 7.51 (d, *J* = 8.6 Hz, 2H, Benzene-H), 7.32 (s, 2H, Benzene-H), 7.26 (d, *J* = 8.1 Hz, 2H, Benzene-H), 7.04 (d, *J* = 8.2 Hz, 2H, Benzene-H), 6.65 (d, *J* = 5.6 Hz, 1H, C5-Pyrimidine-H), 5.07 (s, 3H, CH_2_, OH), 4.43 (d, *J* = 5.7 Hz, 2H, CH_2_,), 2.09 (s, 6H, 2×CH_3_). ^13^C NMR (100 MHz, DMSO-*d*_6_) δ 168.98, 160.77, 159.68, 157.87, 149.58, 145.08, 135.31, 134.97, 133.11, 130.88, 129.05, 128.42, 119.91, 118.59, 114.73, 102.90, 99.33, 69.53, 63.02, 16.55. ESI-MS: *m/z* calcd for C_27_H_24_N_4_O_3_ 451.18 [M − H]^−^, found 451.54 [M − H]^−^.

4-((4-(4-((3-(hydroxymethyl)phenoxy)methyl)-2,6-dimethylphenoxy)pyrimidin-2-yl)amino)benzonitrile (**TF9**)

White solid, yield: 50%, mp: 147–150 °C . ^1^H NMR (400 MHz, DMSO-*d*_6_) δ 10.17 (s, 1H, NH), 8.47 (d, *J* = 5.7 Hz, 1H, C6-Pyrimidine-H), 7.59 (d, *J* = 8.7 Hz, 2H, Benzene-H), 7.51 (d, *J* = 8.4 Hz, 2H, Benzene-H), 7.32 (s, 2H, Benzene-H), 7.27 (t, *J* = 7.9 Hz, 1H, Benzene-H), 7.05 (s, 1H, Benzene-H), 6.94 (d, *J* = 7.8 Hz, 2H, Benzene-H), 6.65 (d, *J* = 5.6 Hz, 1H, C5-Pyrimidine-H), 5.19 (t, *J* = 5.8 Hz, 1H, OH), 5.08 (s, 2H, CH_2_), 4.51 (d, *J* = 5.5 Hz, 2H, CH_2_), 2.09 (s, 6H, 2×CH_3_). ^13^C NMR (100 MHz, DMSO-*d*_6_) δ 168.98, 160.76, 159.68, 158.94, 149.57, 145.08, 144.86, 134.96, 133.13, 130.88, 129.63, 129.05, 119.91, 119.29, 118.60, 113.29, 113.08, 102.91, 99.35, 69.42, 63.24, 16.56. ESI-MS: *m/z* calcd for C_27_H_24_N_4_O_3_ 453.18 [M + H]^+^, found 453.48 [M + H]^+^.

4-((4-(4-((2-(hydroxymethyl)phenoxy)methyl)-2,6-dimethylphenoxy)pyrimidin-2-yl)amino)benzonitrile (**TF10**)

White solid, yield: 45%, mp: 179–182 °C . ^1^H NMR (400 MHz, DMSO-*d*_6_) δ 10.21–10.16 (m, 1H, NH), 8.46 (d, *J* = 5.6 Hz, 1H, C6-Pyrimidine-H), 7.62 (d, *J* = 8.5 Hz, 2H, Benzene-H), 7.49 (d, *J* = 8.6 Hz, 2H, Benzene-H), 7.43 (dd, *J* = 7.5, 1.7 Hz, 1H, Benzene-H), 7.31 (s, 2H, Benzene-H), 7.22 (td, *J* = 7.8, 1.8 Hz, 1H, Benzene-H), 7.08 (d, *J* = 8.2 Hz, 1H, Benzene-H), 6.97 (t, *J* = 7.4 Hz, 1H, Benzene-H), 6.64 (d, *J* = 5.6 Hz, 1H, C5-Pyrimidine-H), 5.34 (s, 1H, OH), 5.14 (s, 2H, CH_2_), 4.60 (s, 2H, CH_2_), 2.10 (s, 6H, 2×CH_3_). ^13^C NMR (100 MHz, DMSO-*d*_6_) δ 169.04, 160.47, 159.54, 155.60, 149.33, 144.99, 135.31, 133.14, 131.24, 130.82, 128.25, 128.01, 127.63, 120.92, 119.84, 118.68, 112.08, 103.03, 99.43, 69.30, 58.43, 16.64.ESI-MS: *m/z* calcd for C_27_H_24_N_4_O_3_ 453.18 [M + H]^+^, found 453.38 [M + H]^+^.

4-((4-(2,6-Dimethyl-4-((pyridin-3-yloxy)methyl)phenoxy)pyrimidin-2-yl)amino) benzonitrile (**TF11**)

White solid, yield: 47%, mp: 237–240 °C . ^1^H NMR (400 MHz, DMSO-*d*_6_) δ 10.16 (s, 1H, NH), 8.46 (d, *J* = 5.6 Hz, 1H, C6-Pyrimidine-H), 7.66 (t, *J* = 2.3 Hz, 1H, Pyridine-H), 7.62 (d, *J* = 5.5 Hz, 1H, Benzene-H), 7.54 (d, *J* = 8.5 Hz, 2H, Benzene-H), 7.39 (s, 2H, Benzene-H), 7.38 (d, *J* = 8.7 Hz, 2H, Benzene-H), 7.32 (dd, *J* = 9.0, 5.4 Hz, 1H, Pyridine-H), 6.96 (dd, *J* = 8.9, 2.7 Hz, 1H, Pyridine-H), 6.65 (d, *J* = 5.6 Hz, 1H, C5-Pyrimidine-H), 5.38 (s, 2H, CH_2_), 2.08 (s, 6H, 2×CH_3_). ^13^C NMR (100 MHz, DMSO-*d*_6_) δ 168.79, 160.82, 159.65, 150.23, 144.99, 134.09, 133.33, 133.26, 133.04, 131.54, 129.48, 127.51, 119.80, 118.48, 102.93, 99.39, 62.23, 16.58. ESI-MS: *m/z* calcd for C_25_H_21_N_5_O_2_ 424.17 [M + H]^+^, found 424.10 [M + H]^+^.

3-((4-((2-((4-cyanophenyl)amino)pyrimidin-4-yl)oxy)-3,5-dimethylbenzyl)oxy) picolinonitrile (**TF12**)

White solid, yield: 41%, mp: 245–248 °C . ^1^H NMR (400 MHz, DMSO-*d*_6_) δ 10.14 (s, 1H, NH), 8.47 (d, *J* = 5.6 Hz, 1H, C6-Pyrimidine-H), 8.36 (d, *J* = 4.5 Hz, 1H, Pyridine-H), 7.97 (d, *J* = 8.7 Hz, 1H, Pyridine-H), 7.76 (dd, *J* = 8.8, 4.5 Hz, 1H, Pyridine-H), 7.61 (d, *J* = 8.5 Hz, 2H, Benzene-H), 7.50 (d, *J* = 8.5 Hz, 2H, Benzene-H), 7.36 (s, 2H, Benzene-H), 6.65 (d, *J* = 5.6 Hz, 1H, C5-Pyrimidine-H), 5.36 (s, 2H, CH_2_), 2.11 (s, 6H, 2×CH_3_). ^13^C NMR (100 MHz, DMSO-*d*_6_) δ 168.91, 160.75, 159.70, 158.34, 149.91, 145.00, 143.70, 133.33, 133.17, 131.18, 129.48, 128.99, 122.69, 122.61, 119.85, 118.58, 115.95, 103.03, 99.41, 70.77, 16.63. ESI-MS: *m/z* calcd for C_26_H_20_N_6_O_2_ 447.16 [M − H]^−^, found 447.24 [M − H]^−^.

5-((4-((2-((4-cyanophenyl)amino)pyrimidin-4-yl)oxy)-3,5-dimethylbenzyl)oxy) nicotinonitrile (**TF13**)

White solid, yield: 52%, mp: 195–198 °C . ^1^H NMR (400 MHz, DMSO-*d*_6_) δ 10.09 (s, 1H, NH), 8.64 (d, *J* = 2.9 Hz, 1H, Pyridine-H), 8.58 (d, *J* = 1.5 Hz, 1H, Pyridine-H), 8.40 (d, *J* = 5.6 Hz, 1H, C6-Pyrimidine-H), 8.05 (t, *J* = 2.3 Hz, 1H, Pyridine-H), 7.51 (d, *J* = 8.5 Hz, 2H, Benzene-H), 7.42 (d, *J* = 8.6 Hz, 2H, Benzene-H), 7.28 (s, 2H, Benzene-H), 6.59 (d, *J* = 5.6 Hz, 1H, C5-Pyrimidine-H), 5.17 (s, 2H, CH_2_), 2.03 (s, 6H, 2×CH_3_). ^13^C NMR (100 MHz, DMSO-*d*_6_) δ 168.91, 160.83, 159.67, 154.64, 149.98, 145.06, 145.01, 143.13, 133.54, 133.10, 131.15, 129.49, 124.45, 119.92, 118.60, 117.21, 109.75, 102.91, 99.34, 70.61, 16.54. ESI-MS: *m/z* calcd for C_26_H_20_N_6_O_2_ 447.16 [M − H]^−^, found 447.27 [M − H]^−^.

4-((4-(4-(((6-fluoropyridin-3-yl)oxy)methyl)-2,6-dimethylphenoxy)pyrimidin-2-yl)amino)benzonitrile (**TF14**)

White solid, yield: 61%, mp: 185–188 °C . ^1^H NMR (400 MHz, DMSO-*d*_6_) δ 10.16 (s, 1H, NH), 8.47 (d, *J* = 5.6 Hz, 1H, C6-Pyrimidine-H), 8.04 (dd, *J* = 3.1, 1.8 Hz, 1H, Pyridine-H), 7.74 (ddd, *J* = 9.4, 6.7, 3.1 Hz, 1H, Pyridine-H), 7.58 (d, *J* = 8.6 Hz, 2H, Benzene-H), 7.49 (d, *J* = 8.6 Hz, 2H, Benzene-H), 7.34 (s, 2H, Benzene-H), 7.16 (dd, *J* = 8.9, 3.4 Hz, 1H, Pyridine-H), 6.65 (d, *J* = 5.6 Hz, 1H, C5-Pyrimidine-H), 5.17 (s, 2H, CH_2_), 2.10 (s, 6H, 2×CH_3_). ^13^C NMR (100 MHz, DMSO-*d*_6_) δ 168.93, 160.80, 159.67, 158.86, 156.58, 153.59, 153.55, 149.81, 145.06, 134.15, 133.86, 133.70, 133.11, 131.04, 129.26, 128.98, 128.90, 119.91, 118.59, 110.50, 110.10, 102.89, 99.32, 70.73, 16.54. ESI-MS: *m/z* calcd for C_25_H_20_FN_5_O_2_ 442.16 [M + H]^+^, found 442.61 [M + H]^+^.

4-((4-(2,6-Dimethyl-4-((4-nitrophenoxy)methyl)phenoxy)pyrimidin-2-yl)amino) benzonitrile (**TF15**)

White solid, yield: 71%, mp: 233–236 °C . ^1^H NMR (400 MHz, DMSO-*d*_6_) δ 10.20 (s, 1H, NH), 8.48 (d, *J* = 5.6 Hz, 1H, C6-Pyrimidine-H), 8.25 (d, *J* = 8.8 Hz, 2H, Benzene-H), 7.59 (d, *J* = 8.6 Hz, 2H, Benzene-H), 7.49 (d, *J* = 8.5 Hz, 2H, Benzene-H), 7.37 (s, 2H, Benzene-H), 7.31 (d, *J* = 8.9 Hz, 2H, Benzene-H), 6.67 (d, *J* = 5.6 Hz, 1H, C5-Pyrimidine-H), 5.27 (s, 2H, CH_2_), 2.11 (s, 6H, 2×CH_3_). ^13^C NMR (100 MHz, DMSO-*d*_6_) δ 168.91, 164.18, 160.80, 159.66, 149.93, 145.08, 141.45, 133.73, 133.06, 131.11, 129.33, 126.36, 119.92, 118.57, 115.71, 102.86, 99.30, 70.56, 16.53. ESI-MS: *m/z* calcd for C_26_H_21_N_5_O_4_ 468.16 [M + H]^+^, found 468.19 [M + H]^+^.

(4-((4-((2-((4-cyanophenyl)amino)pyrimidin-4-yl)oxy)-3,5-dimethylbenzyl)oxy) phenyl)carbamic acid tert-butyl ester (**TF16**)

White solid, yield: 73%, mp: 184–187 °C . ^1^H NMR (400 MHz, DMSO-*d*_6_) δ 10.15 (s, 1H, NH), 9.14 (s, 1H, NH), 8.46 (d, *J* = 5.7 Hz, 1H, C6-Pyrimidine-H), 7.58 (d, *J* = 8.7 Hz, 2H, Benzene-H), 7.51 (d, *J* = 8.7 Hz, 2H, Benzene-H), 7.38 (d, *J* = 8.5 Hz, 2H, Benzene-H), 7.31 (s, 2H, Benzene-H), 7.02–6.96 (m, 2H, Benzene-H), 6.64 (d, *J* = 5.7 Hz, 1H, C5-Pyrimidine-H), 5.01 (s, 2H, CH_2_), 2.09 (s, 6H, 2×CH_3_), 1.47 (s, 9H, 3×CH_3_). ^13^C NMR (100 MHz, DMSO-*d*_6_) δ 168.97, 160.77, 159.67, 154.19, 153.40, 149.57, 145.07, 134.99, 133.34, 133.12, 130.85, 129.14, 120.11, 119.90, 118.60, 115.14, 102.90, 99.32, 79.13, 69.75, 28.65, 16.54. ESI-MS: *m/z* calcd for C_31_H_31_N_5_O_4_ 538.24 [M + H]^+^, found 538.16 [M + H]^+^. 

#### 3.1.2. Preparation Method of **TF17**

**TF16** (0.2 g, 0.37 mmol) was dissolved in 5 mL dichloromethane. Following the addition of the trifluoroacetic acid (2 mL), the mixture was stirred for 5 hours at room temperature. After the reaction was finished (monitored by TLC), the reaction solution was mixed with water (10 mL) and adjusted pH value to 9–10 with saturated sodium bicarbonate aqueous. Dichloromethane (3 × 20 mL) was used to extract the reaction solution after adding saturated sodium chloride solution (30 mL). Then, the organic layer was dried with anhydrous sodium sulfate. Flash column chromatography was used to separate **TF17** from the mixture with ethyl acetate and petroleum ether.

4-((4-(4-((4-Aminophenoxy)methyl)-2,6-dimethylphenoxy)pyrimidin-2-yl)amino) benzonitrile (**TF17**)

White solid, yield: 65%, mp: 241–244 °C . ^1^H NMR (400 MHz, DMSO-*d*_6_) δ 10.17 (s, 1H, NH), 9.35 (s, 2H, NH_2_), 8.47 (d, *J* = 5.7 Hz, 1H, C6-Pyrimidine-H), 7.56 (d, *J* = 8.7 Hz, 2H, Benzene-H), 7.50 (d, *J* = 8.6 Hz, 2H, Benzene-H), 7.32 (s, 2H, Benzene-H), 7.21 (d, *J* = 8.5 Hz, 2H, Benzene-H), 7.14 (d, *J* = 8.5 Hz, 2H, Benzene-H), 6.66 (d, *J* = 5.7 Hz, 1H, C5-Pyrimidine-H), 5.09 (s, 2H, CH_2_), 2.09 (s, 6H, 2×CH_3_). ^13^C NMR (100 MHz, DMSO-*d*_6_) δ 168.95, 160.83, 159.65, 157.18, 149.71, 145.09, 134.59, 133.11, 130.96, 129.20, 127.78, 123.35, 119.95, 118.57, 116.11, 102.85, 99.31, 69.97, 16.54. ESI-MS: *m/z* calcd for C_26_H_23_N_5_O_2_ 438.19 [M + H]^+^, found 438.57 [M + H]^+^.

### 3.2. In Vitro Anti-HIV Assay 

Evaluation of the anti-HIV activity and cytotoxicity of the target compounds was performed by utilizing the MTT method in MT-4 cells described previously [37]. Stock solutions (10 × final concentration) of test compounds were added in 25 μL volumes to two series of triplicate wells to allow simultaneous evaluation of their effects on mock- and HIV-infected cells at the beginning of each experiment. Serial five-fold dilutions of test compounds were prepared directly in flat-bottomed 96-well microtiter trays by adding 100 μL medium to the 25 μL stock solution and transferring 25 μL of this solution to another well that contained 100 μL medium using a Biomek 3000 robot (Beckman Instruments, Fullerton, CA). Each sample includes untreated control HIV- and mock-infected cell samples. WT HIV-1 strain (IIIB), HIV-1 drug-resistant strains including L100I, K103N, Y181C, Y188L, E138K, K103N/Y181C, F227L/V106A or HIV-2 strain (ROD) stock (50 μL) at 100–300 CCID50 (50% cell culture infectious dose) or culture medium was added to either the infected or mock-infected wells of the microtiter tray. Mock-infected cells were used to evaluate the effect of test compounds on uninfected cells to evaluate their cytotoxicity. Exponentially growing MT-4 cells were centrifuged for 5 min at 1000 rpm, and the supernatant was discarded. The MT-4 cells were resuspended at 6 × 105 cells/mL and then transferred 50 μL volumes to the microtiter tray wells. After infection five days, the viability of mock- and HIV-infected cells was examined spectrophotometrically by the MTT assay. The 50% cytotoxic concentration (CC_50_) was defined as the concentration of the test compound that reduced the viability of the mock-infected MT4 cells by 50%. The 50% effective concentration (EC_50_) was defined as the concentration of the test compound achieving 50% protection from the cytopathic effect of the virus in infected cells.

### 3.3. HIV-1 RT Inhibition Assay 

An HIV-1 reverse transcriptase (RT) assay kit produced by Roche was used for the RT inhibition assay. All the reagents for performing the RT reaction came from the kit, and the ELSIA procedures for RT inhibition assay were conducted following the description in the kit protocol [38]. In brief, the reaction mixture containing template/primer complex, viral nucleotides (dNTPs) and HIV-1 reverse transcriptase (RT) enzyme in the incubation buffer with or without inhibitors was incubated for 1 h at 37 °C. Subsequently, the reaction mixture was transferred to a streptavidine-coated microtiter plate (MTP) and incubated for another 1 h at 37 °C to ensure the retranscriptional cDNA chain that consisted biotin labeled dNTPs bound to streptavidine. Then used, a washing buffer was used to remove the unbound dNTPs, and an anti-DIG-POD working solution was added. After incubation for 1 h at 37 °C, the DIG-labeled dNTPs incorporated in cDNA were bound to the anti-DIG-POD antibody. The unbound anti-DIG-PODs were removed, and the peroxidesubstrate (ABST) solution was added to the MTPs. A colored reaction proceeds during the cleavage of the substrate catalyzed by POD. The absorbance of the sample was determined at O.D. 405 nm using a microtiter plate ELISA reader. The percentage inhibitory activity of RT inhibitors was calculated by the formula given below:

% Inhibition = [O.D. value with RT but without inhibitors − O.D. value with RT and inhibitors]/[O.D. value with RT and inhibitors − O.D. value without RT and inhibitors].

The IC_50_ values corresponded to the concentrations of the test compounds required to inhibit the incorporation of biotin-dUTP into RT by 50%.

### 3.4. MD Simulation Methods 

Ligand preparation: The ligand structure **TF2** was generated with Chemdraw (version 20.0), and the structure was then transferred to 3D molecules using Chem3D (version 20.0), which was then prepared with the LigPrep module of Schrodinger suites. The ligands were prepared with an OPLS4 force field, and the ionization states were generated via Epik at pH 7.0. A maximum of 32 conformations were generated per ligand, and the best-scoring conformation was used for docking.

Ligand Docking: The co-crystal structures of HIV-1 WT RT in complex with RPV (PDB code:2ZD1) [22], and HIV-1 K103N RT in complex with RPV (PDB code:3MEG) [27] were prepared using the Protein Preparation Wizard module of Schrodinger suites (Release 2022-1). All original hydrogens were removed and re-added. The protein bond order was reassigned with the CCD database, and H_2_O molecules were deleted. The protonation state for the protein complex was set to pH 7.0. Furthermore, the missing side chains were rebuilt using prime. The hydrogen bond order was reassigned with PROPKA, and restrained minimization was carried out with the OPLS4 force field. The generated protein complex structures were then used for docking.

Induced-fit docking (Schrodinger suites, Release 2022-1) was performed to investigate the binding poses of **TF2** with WT and K103N RT, respectively. Standard protocol was used, and a maximum of 20 docking poses were generated per ligand. The nonnucleoside reverse transcriptase inhibitor (NNRTI) RPV binding pocket was selected as the binding pocket. The other parameters were set as default. The binding conformations of each analogue with the best IFDs core were selected for analysis and next step MD simulations (Figure S1). 

Molecular Dynamic simulations: The input file for MD was the output file from the best docked position of the Schrödinger Induced Fit Docking Panel [40]. A 500 ns molecular dynamic simulation was run using the Desmond module of Schrödinger Suite 2022-1 with an OPLS4 force field. Using the TIP3P water model and Desmond’s system builder module, the protein-ligand complex was solved, and an orthorhombic simulation box with a buffer distance of 10 Å between its edge and the atoms of the complex was created. A sufficient number of counter-ions were added to the system to neutralize it, and 0.15 M NaCl was added to the simulation box to preserve the isotonic condition. Energy minimization was applied to the model up until a gradient threshold of 25 kcal/mol/Å was reached at 300 K and 1 bar of pressure using NPT ensemble class. The trajectory was documented as the MD simulation was running, and using Desmond’s Simulation Event Analysis tool, Protein-Ligand interactions, Protein-Ligand fluctuations, and contacts with different amino acids were used to gauge the stability of the complex.

Free ligand-binding energy calculation (MM-GBSA): Free ligand-binding energy calculation (MM-GBSA) [41]: The MM/GBSA calculation (Schrödinger Release 2022-1) was performed to estimate the binding free energy of ligands when binding the various RTs.

## 4. Conclusions

In summary, based on the X-ray crystallographic analysis, to explore the primer grip region and target the conserved residues, we have designed and synthesized 17 DAPY derivatives with -CH_2_O- as the linker. According to the biological evaluation results, all compounds (EC_50 =_ 7.6 nM ~ 199 nM) exhibited potent activity against HIV-1 WT strain, **TF2** (EC_50_ = 7.6 nM), **TF4** (EC_50_ = 7.8 nM) and **TF12** (EC_50_ = 7.8 nM) showed the most potent activity, which was better than **NVP** (EC_50_ = 122.6 nM). Notably, **TF2** (CC_50_ > 279,329.6 nM) showed low cytotoxicity. And for the tested HIV-1 mutant strains, **TF2** (EC_50 (K103N)_ = 28.1 nM, EC_50 (E138K)_ = 44.0 nM, EC_50 (Y181C)_ = 139.3 nM) was the most potent compound against HIV-1 K103N, E138K and Y181C mutant strains. Moreover, the WT HIV-1 RT enzyme inhibitory assay indicated that the target of these novel compounds was RT. Furthermore, detailed SARs of compounds and molecular dynamics simulation of **TF2** can provide insight into the binding modes of compounds, and the further structural optimization of the compound can be guided accordingly. The molecular dynamics simulation studies of **TF2** showed that the newly introduced *p*-cyanophenyl moiety reached the primer grip region, but no interaction with the key amino acid here was observed. The primer grip region has yet to be explored, and the development of novel NNRTI drugs with improved tolerability and resistance profiles is still required.

## Figures and Tables

**Table 1:** The anti-HIV-1(IIIB) activity and cytotoxicity of **TF1-17**.

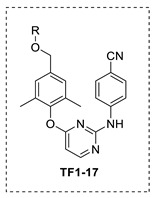					
Compounds	R	EC_50_ *^a^*		CC_50_ *^b^* (nM)	SI *^c^* (IIIB)
		IIIB (nM)	ROD (nM)		
**TF1**	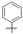	17.1 ± 2.3	>295,865.0	>295,865.0	>17,326.7
**TF2**	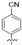	7.6 ± 0.6	>279,329.6	>279,329.6	>36,610.9
**TF3**	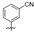	24.5 ± 4.1	>5522.3	5522.3 ± 296.6	225.2
**TF4**	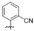	7.8 ± 1.0	>5293.0	5293.0 ± 355.6	674.4
**TF5**	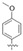	21.7 ± 3.6	>253,718.1	253,718.1 ± 5720.2	11,681.3
**TF6**	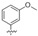	35.3 ± 4.1	>6896.1	6896.1 ± 249.1	195.6
**TF7**	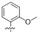	25.5 ± 8.4	>8152.2	8152.2 ± 547.6	319.6
**TF8**	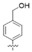	42.8 ± 5.6	>2917.9	2917.9 ± 271.5	68.1
**TF9**	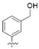	30.5 ± 4.0	>3458.6	3458.6 ± 375.4	113.3
**TF10**	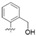	32.2 ± 3.3	>4804.6	4804.6 ± 341.7	149.1
**TF11**	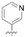	199.0 ± 13.0	>14,827.6	14,827.6 ± 919.6	74.5
**TF12**	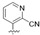	7.8 ± 0.8	>2462.8	2462.8 ± 197.9	316.9
**TF13**	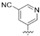	19.4 ± 3.4	>4772.8	4772.8 ± 1561.8	246.4
**TF14**	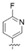	23.6 ± 3.8	>24,438.7	24,438.7 ± 6675.8	1034.6
**TF15**	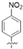	23.1 ± 3.1	>215,001.1	215,001.1 ± 20,337.8	9303.7
**TF16**	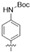	45.3 ± 14.1	>6196.6	6196.6 ± 220.4	136.7
**TF17**	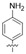	32.5 ± 1.7	>5489.9	5489.9 ± 328.0	168.8
**AZT**	——	27.3 ± 3.5	23.3 ± 2.1	>7483.6	>274.0
**NVP**	——	122.6 ± 7.3	-	>15,020.7	>122.5
**EFV**	——	2.4 ± 0.3	-	>6335.5	>2689.1
**ETR**	——	2.9 ± 0.4	-	>4594.6	>1578.9
**RPV** *^d^*	——	1.00 ± 0.27	-	3.98 (µM)	3989

*^a^* EC_50_: concentration of compounds protecting 50% of MT-4 cells from lesions caused by HIV-1 virus infection. *^b^* CC_50_: concentration of compounds causing 50% of HIV-uninfected MT-4 cells. *^c^* SI: selectivity index, CC_50_/EC_50_. *^d^* Used for comparison [5]. Standard deviation values were obtained from five parallel experiments.

**Table 2:** The activity of anti-HIV-1 mutant strains of **TF1–17**.

Compounds	EC_50_ *^a^* (nM)						
	L100I	K103N	Y181C	Y188L	E138K	F227L + V106A	RES056
**TF1**	221.3 ± 53.5	50.7 ± 9.6	515.3 ± 60.4	1519.8 ± 92.1	76.5 ± 9.4	1710.6 ± 581.4	6853.1 ± 1172.2
**TF2**	117.0 ± 49.5	28.1 ± 7.1	139.3 ± 34.9	1390.3 ± 118.8	44.0 ± 2.9	1136.6 ± 346.5	> 279,329.6
**TF3**	116.0 ± 5.8	48.4 ± 7.5	245.0 ± 39.6	894.7 ± 90.2	87.7 ± 8.5	755.4 ± 155.6	1543.5 ± 102.5
**TF4**	314.9 ± 30.7	75.9 ± 13.9	482.3 ± 42.6	> 5296.1	103.1 ± 6.8	1279.5 ± 303.3	> 5296.1
**TF5**	126.1 ± 12.6	44.5 ± 4.9	267.8 ± 28.9	963.7 ± 98.1	65.3 ± 10.0	1406.4 ± 569.6	> 253,718.1
**TF6**	156.0 ± 16.8	91.2 ± 14.4	386.7 ± 87.4	1391.7 ± 83.6	149.7 ± 0.7	1096.7 ± 281.1	> 6896.1
**TF7**	451.9 ± 70.5	164.1 ± 5.5	790.6 ± 62.9	> 8152.2	180.7 ± 10.7	1224.1 ± 179.3	> 8152.2
**TF8**	328.9 ± 56.7	110.2 ± 4.6	667.2 ± 78.0	885.5 ± 20.1	147.5 ± 4.6	853.3 ± 81.4	1418.6 ± 9.7
**TF9**	308.1 ± 46.3	119.6 ± 18.2	499.2 ± 44.6	1095.3 ± 95.7	147.1 ± 12.0	999.8 ± 29.6	1586.6 ± 87.5
**TF10**	634.6 ± 28.2	158.4 ± 18.6	765.6 ± 22.5	> 4804.6	178.8 ± 26.9	1487.4 ± 33.9	> 4804.6
**TF11**	9574.0 ± 22.2	444.1 ± 68.6	> 14,827.6	> 14,827.6	≥ 3024.0	> 14,827.6	> 14,827.6
**TF12**	245.6 ± 54.6	34.7 ± 8.8	384.5 ± 75.5	≥ 1560.8	46.2 ± 7.1	742.4 ± 145.1	> 2462.8
**TF13**	125.3 ± 5.9	28.0 ± 2.5	265.2 ± 24.6	1053.3 ± 66.8	57.7 ± 11.6	1650.5 ± 19.9	1182.9 ± 68.2
**TF14**	185.1 ± 11.6	36.7 ± 3.7	285.5 ± 52.1	773.6 ± 39.2	67.4 ± 8.1	999.4 ± 125.8	1192.8 ± 182.2
**TF15**	105.2 ± 15.2	46.3 ± 9.7	195.9 ± 25.4	1079.6 ± 99.4	50.0 ± 4.4	1655.9 ± 448.3	1801.7 ± 68.5
**TF16**	715.2 ± 183.5	131.7 ± 28.4	1274.9 ± 153.7	> 6196.6	245.5 ± 30.4	≥ 1618.2	> 6196.6
**TF17**	226.5 ± 28.9	65.3 ± 7.6	552.5 ± 62.5	905.1 ± 147.4	151.9 ± 10.3	747.1 ± 72.2	> 5.490
**AZT**	10.0 ± 1.2	22.8 ± 1.1	25.1 ± 3.0	19.4 ± 2.9	30.1 ± 3.0	14.8 ± 1.1	25.1 ± 1.8
**NVP**	1856.7 ± 364.6	7495.1 ± 866.8	10,113.7 ± 850.9	≥ 13,781.4	149.4 ± 12.0	> 15,020.7	> 15,020.7
**EFV**	35.0 ± 4.7	95.1 ± 9.1	5.5 ± 0.5	239.6 ± 30.4	4.4 ± 0.3	200.8 ± 17.3	183.8 ± 22.0
**ETR**	5.9 ± 1.0	3.0 ± 0.3	11.1 ± 1.3	18.1 ± 3.2	6.3 ± 1.2	20.4 ± 7.8	29.5 ± 4.8
**RPV** *^b^*	1.54 ± 0.00	1.31 ± 0.36	4.73 ± 0.48	79.4 ± 0.77	5.75 ± 0.11	5.75 ± 0.11	10.7 ± 7.96

*^a^* EC_50_: concentration of compounds protecting 50% of MT-4 cells from lesions caused by HIV-1 virus infection. *^b^* Used for comparison [5]. Standard deviation values were obtained from five parallel experiments.

**Table 3:** Resistance fold for K103N mutant strain of **TF1–17**.

Compounds	Resistance Folds *^a^*	Compds	Resistance Folds *^a^*
	K103N		K103N
**TF1**	3.00	**TF10**	4.94
**TF2**	3.68	**TF11**	2.23
**TF3**	1.92	**TF12**	4.49
**TF4**	9.74	**TF13**	1.47
**TF5**	2.00	**TF14**	1.54
**TF6**	2.60	**TF15**	2.00
**TF7**	6.31	**TF16**	2.93
**TF8**	2.56	**TF17**	1.97
**TF9**	3.87	**EFV**	47.5

*^a^* Resistance fold: RF, EC_50 (K103N)_/EC_50 (WT)_.

**Table 4:** The activity of **TF1**-**TF17** against HIV-1 WT RT.

Compds	IC_50_ *^a^* (μM)	Compds	IC_50_ *^a^* (μM)
**TF1**	0.062 ± 0.000	**TF11**	0.483 ± 0.03
**TF2**	0.055 ± 0.001	**TF12**	0.038 ± 0.004
**TF3**	0.060 ± 0.000	**TF13**	0.059 ± 0.001
**TF4**	0.050 ± 0.002	**TF14**	0.041 ± 0.003
**TF5**	0.054 ± 0.004	**TF15**	0.063 ± 0.001
**TF6**	0.052 ± 0.004	**TF16**	0.160 ± 0.001
**TF7**	0.073 ± 0.003	**TF17**	0.057 ± 0.003
**TF8**	0.041 ± 0.001	**NVP**	0.568 ± 0.063
**TF9**	0.053 ± 0.000	**EFV**	0.013 ± 0.001
**TF10**	0.055 ± 0.000	**ETR**	0.011 ± 0.000 *^b^*

*^a^* IC_50_: Concentration of compounds needed to prevent 50% of biotin deoxyuridine triphosphate (biotin-dUTP) from being incorporated into HIV-1 WT RT. *^b^* Used for comparison [5].

**Table 5:** MM/GBSA energy calculations.

Binding Energies ^a^ (kcal/mol)	TF2 → NNIBP (WT)	TF2 → NNIBP (K103N)
Δ*G_Hbond_*	−1.1 ± 0.3	−0.9 ± 0.3
Δ*G_bind_*	−113.8 ± 3.8	−109.0 ± 4.5

^a^ Δ*G_Hbond_* = Hydrogen-bonding correction; Δ*G_bind_* = total binding free energy.

## Data Availability

Data is contained within the article.

## Supplementary Materials

The following supporting information can be downloaded at: https://www.mdpi.com/article/10.3390/ph15111438/s1, Figure S1, ^1^H and ^13^C NMR spectra of **TF1–17**.
