# Supplementary material for: Identification of Novel Diarylpyrimidines as Potent HIV-1 Non-Nucleoside Reverse Transcriptase Inhibitors by Exploring the Primer Grip Region
Source: Pharmaceuticals (Basel). 2022 Nov 19;15(11):1438. doi: 10.3390/ph15111438 (PMC9697031; doi:10.3390/ph15111438)

Supporting Information

1. Ligand Docking Result

Induced-fit docking (Schrodinger suites, Release 2022-1) were performed to investigate the binding poses of **TF2** with WT and K103N RT, respectively. Standard protocol was used and maximum 20 docking poses were generated per ligands. The non-nucleoside reverse transcriptase inhibitor (NNRTI) RPV binding pocket was selected as the binding pocket. The other parameters were set as default. The binding conformations of each analogue with best IFDs core were selected for analysis and next step MD simulations. The docking results showed that **TF2** and the co-crystallised inhibitor RPV share the same chemical scaffold (**Figure 1 SA** and **SB**) and adopted a common pose (U shape) in the NNRTI-binding site of WT and K103N RT (**Figure1 SC** and **SD**). And they were used as starting structures for the MD simulation.

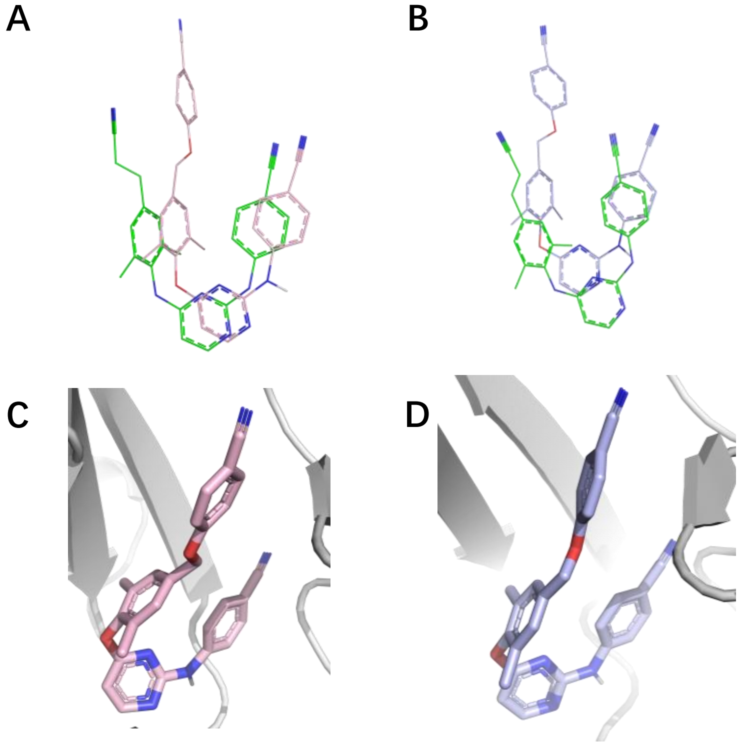

Figure S1. A) Superposition of **TF2** (light pink) and the co-crystallised compound RPV (green) with HIV-1 WT RT. **B**) Superposition of **TF2** (light blue) and the co-crystallised compound RPV (green) with HIV-1 K103N RT. The starting structure of **TF2** for MD simulation with the x-ray structure **C**) 2zd1 (HIV-1 WT RT) and **D**) 3meg (HIV-1 K103N RT), respectively.

2. ^{1}H and ^{13}C NMR spectra of TF1-17

^{1}H and ^{13}C NMR spectra of TF1

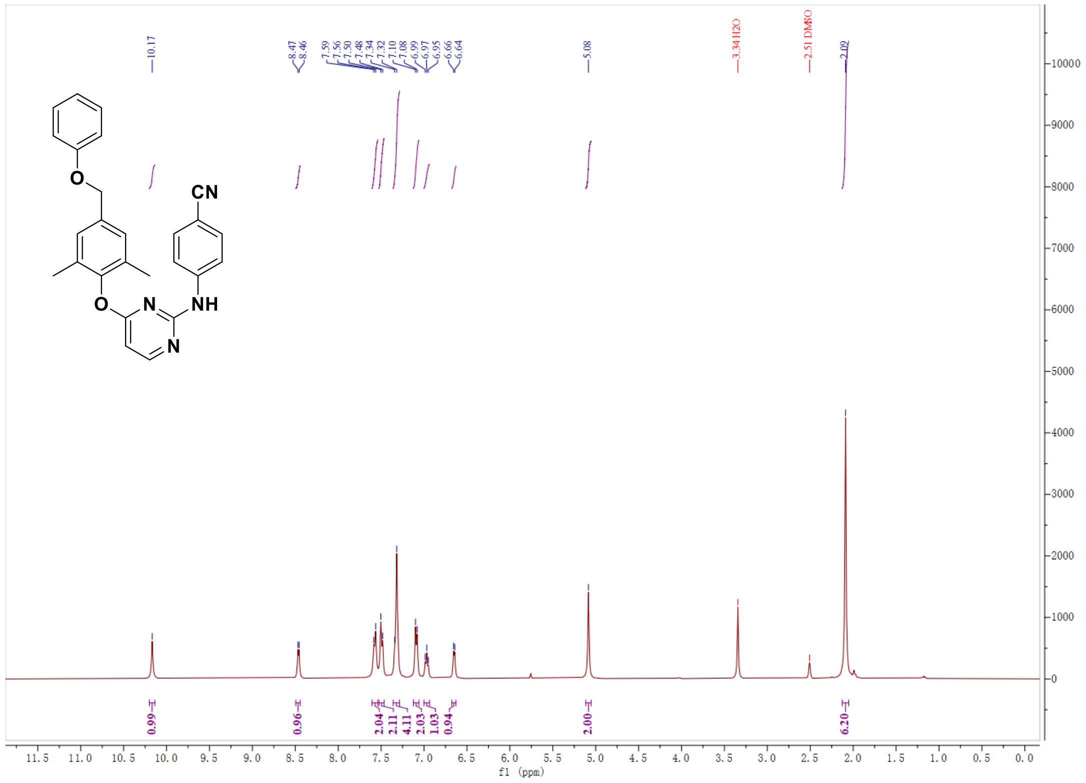

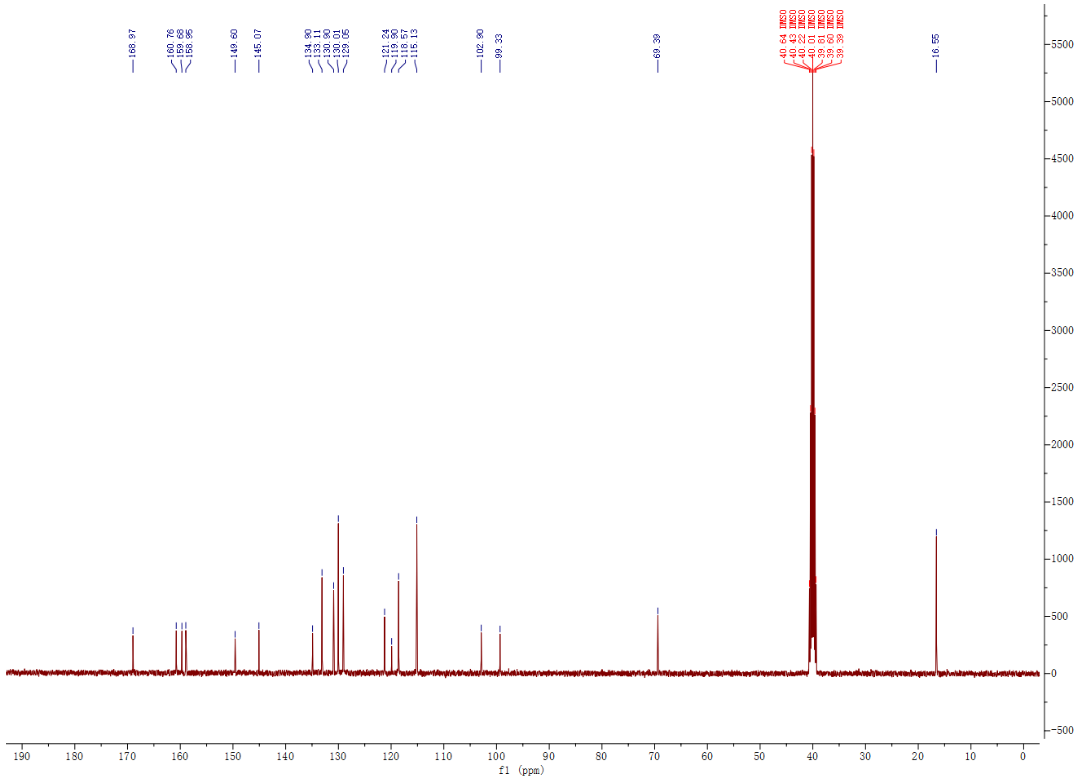

^{1}H and ^{13}C NMR spectra of TF2

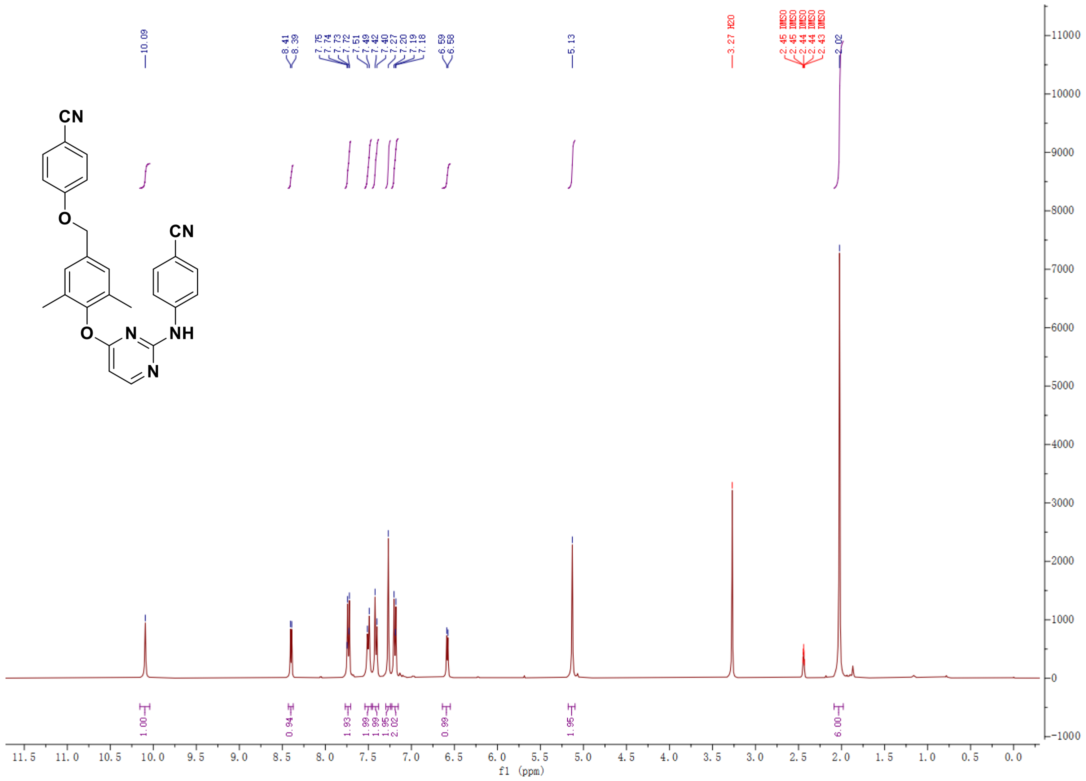

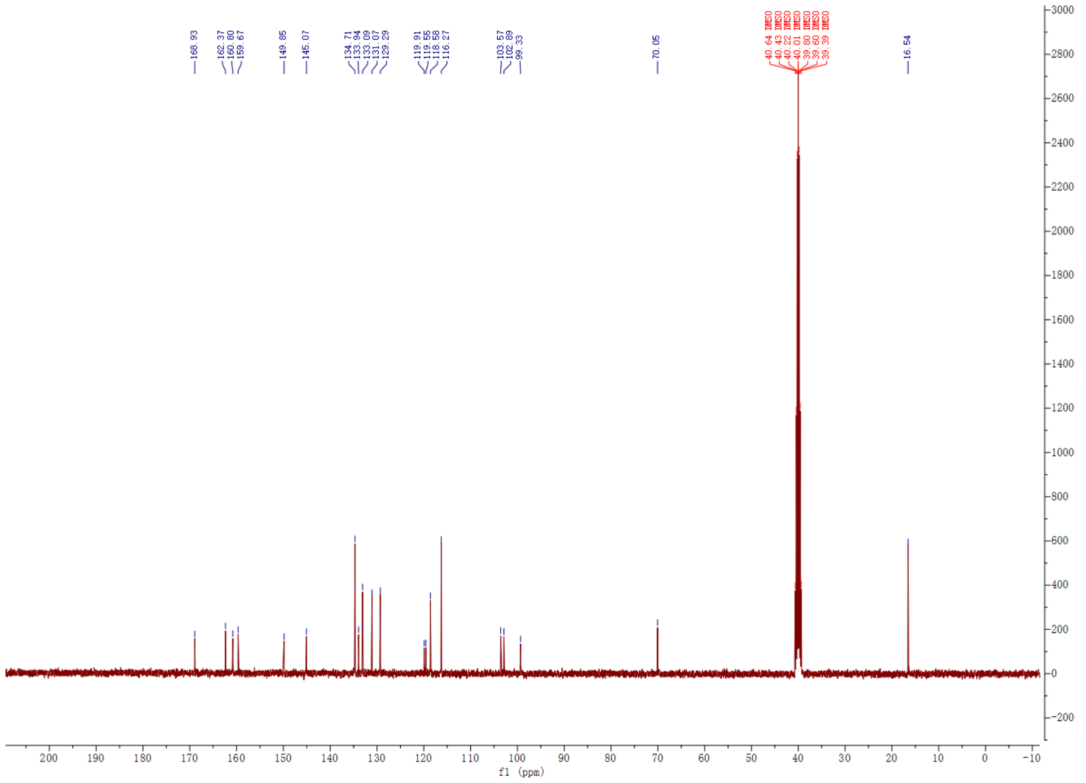

^{1}H and ^{13}C NMR spectra of TF3

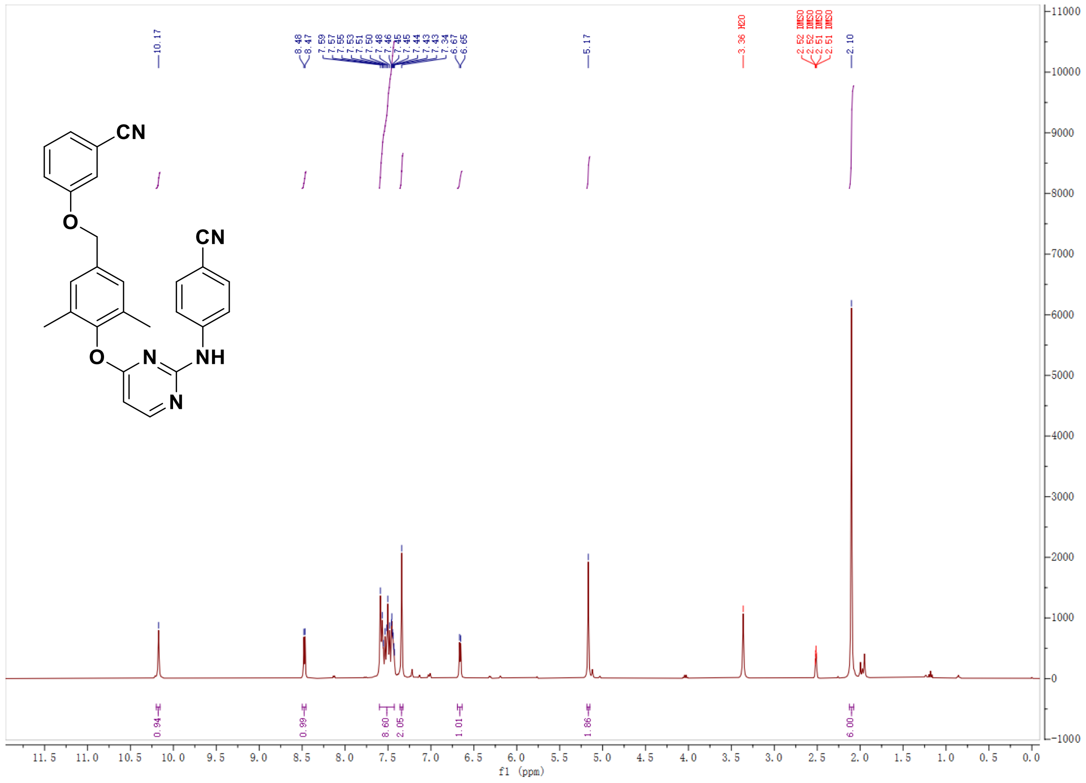

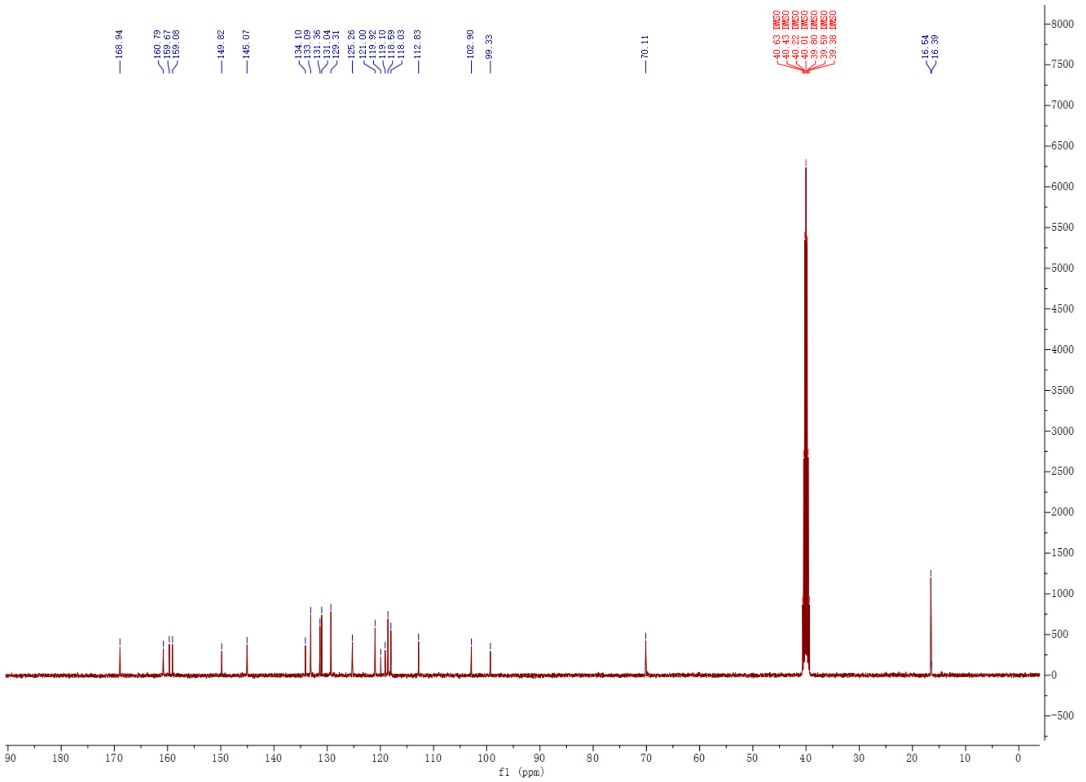

^{1}H and ^{13}C NMR spectra of TF4

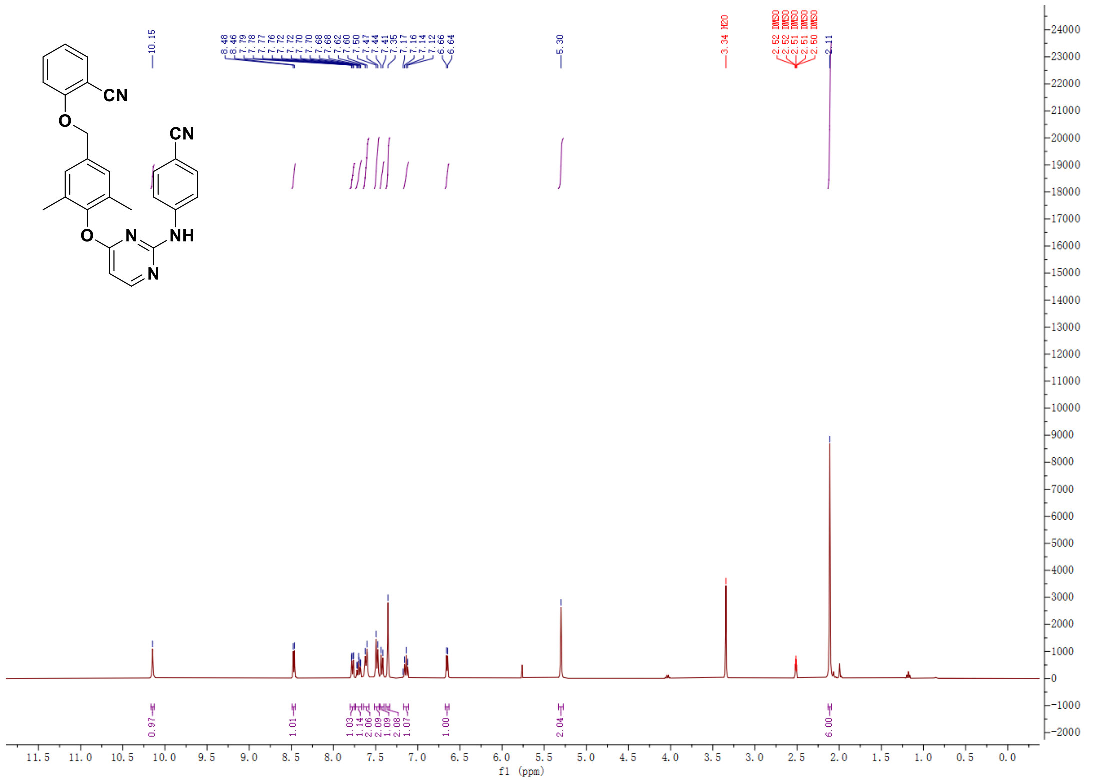

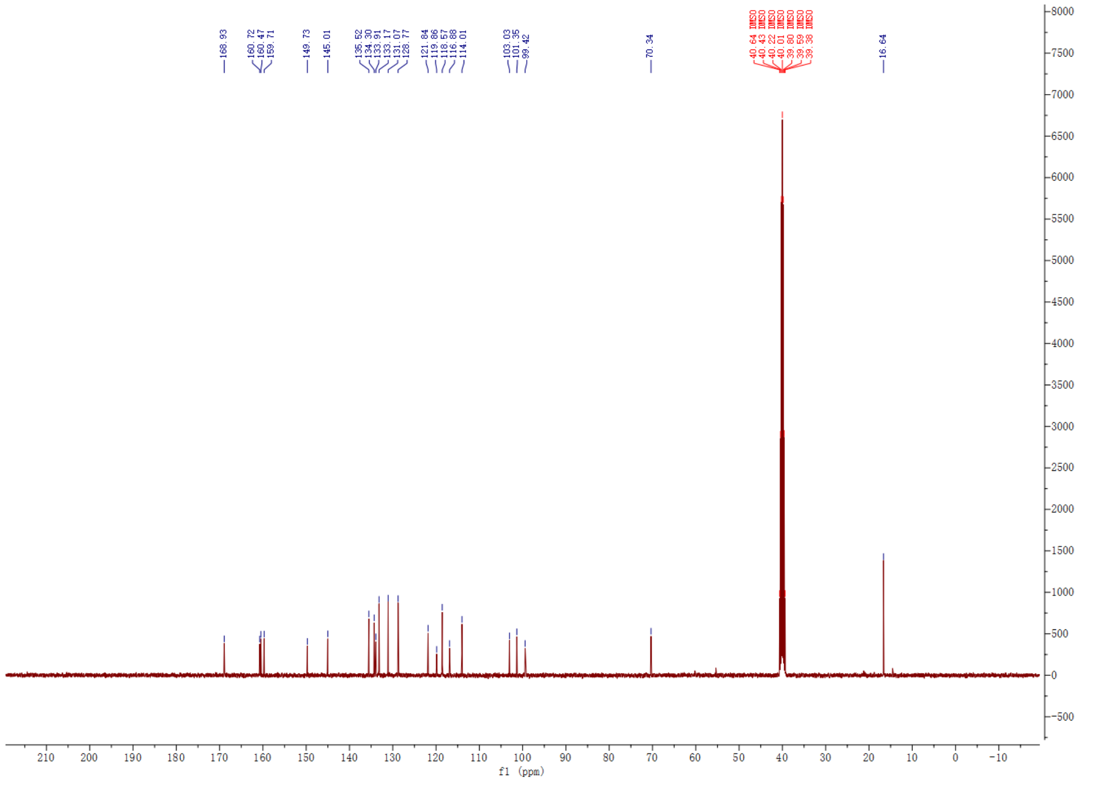

^{1}H and ^{13}C NMR spectra of TF5

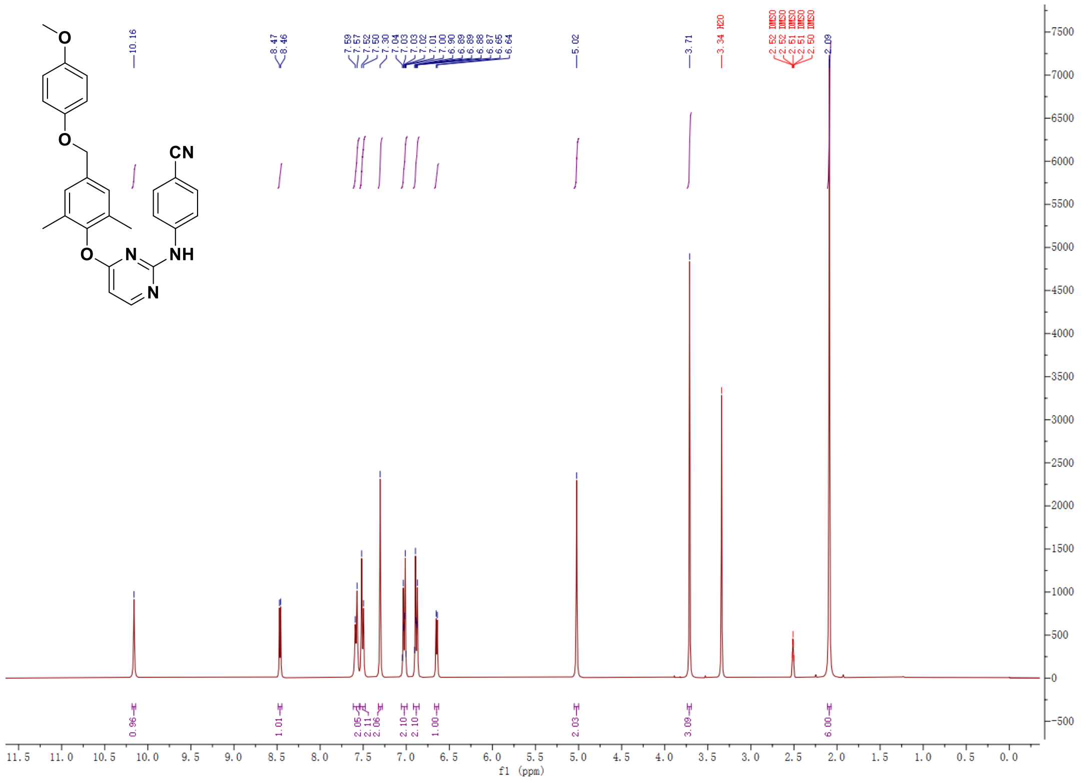

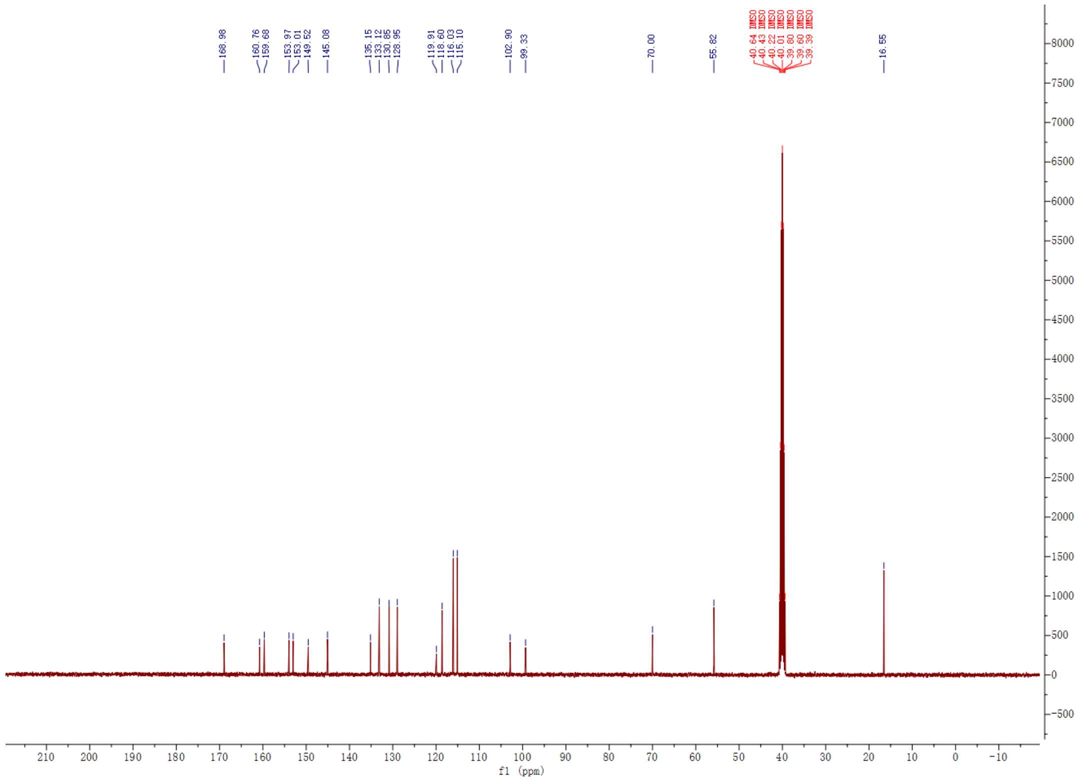

^{1}H and ^{13}C NMR spectra of TF6

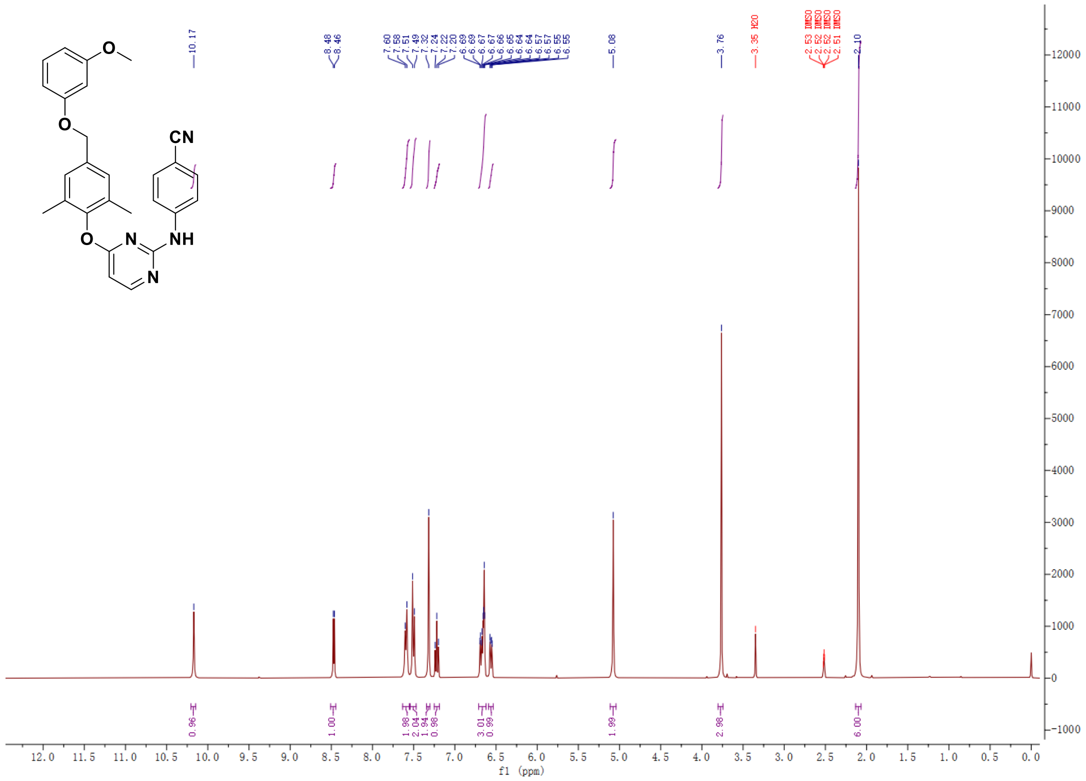

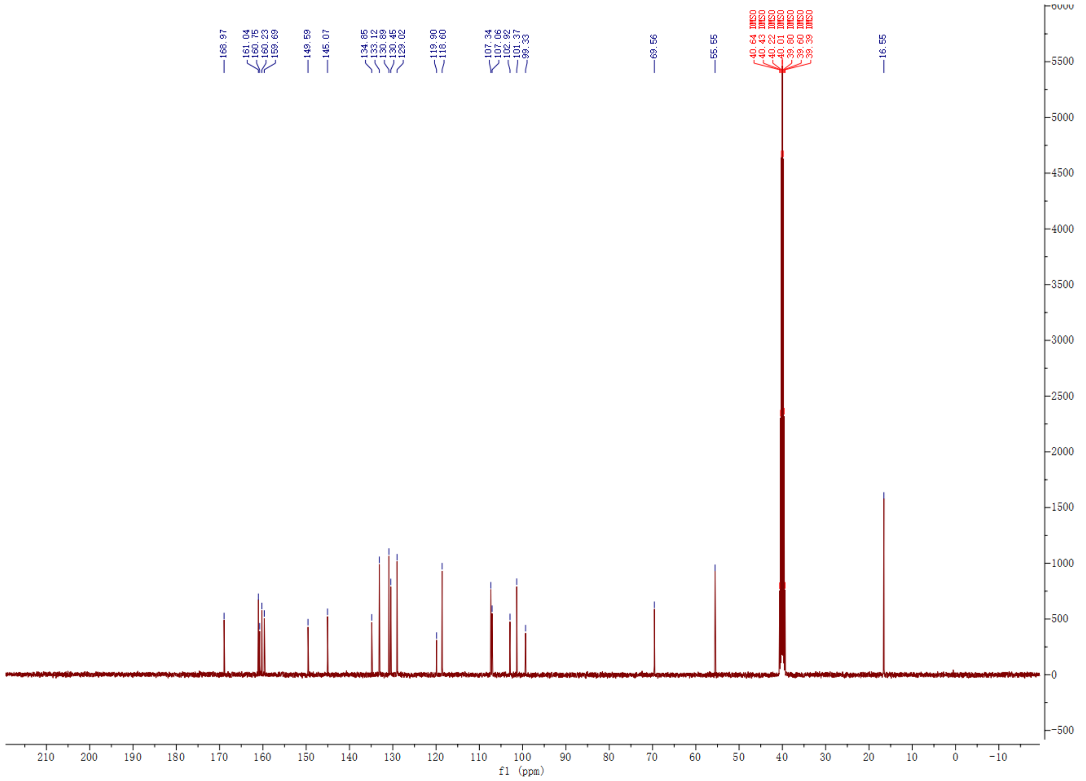

^{1}H and ^{13}C NMR spectra of TF7

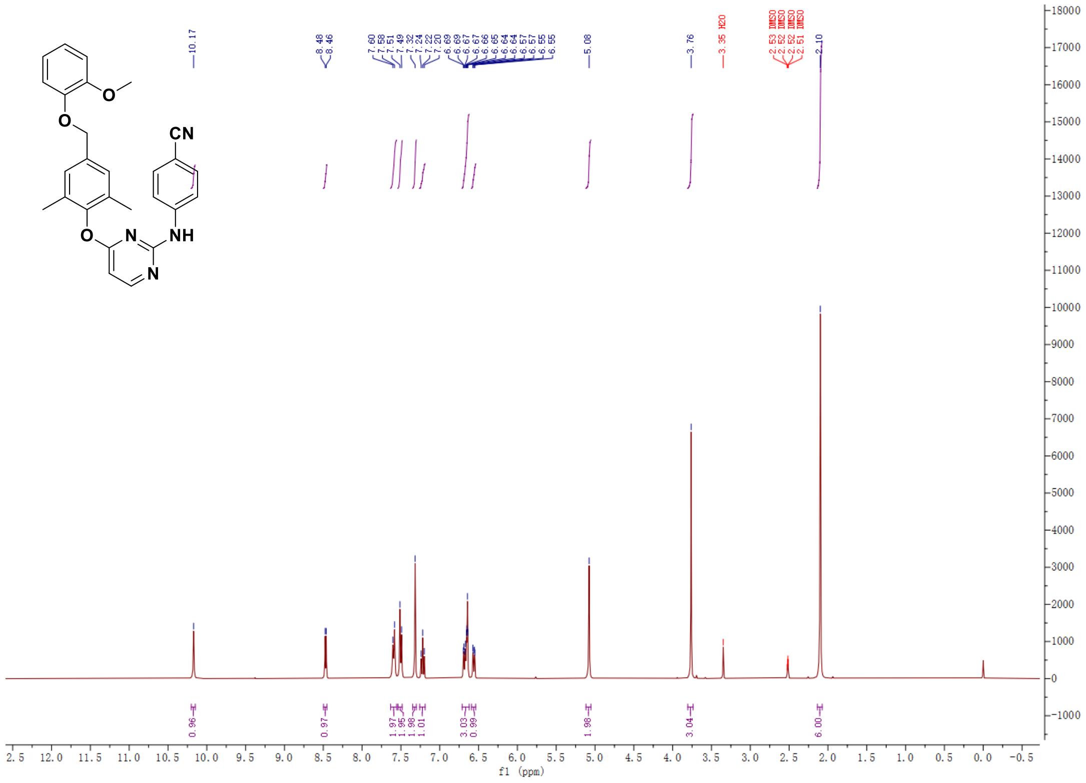

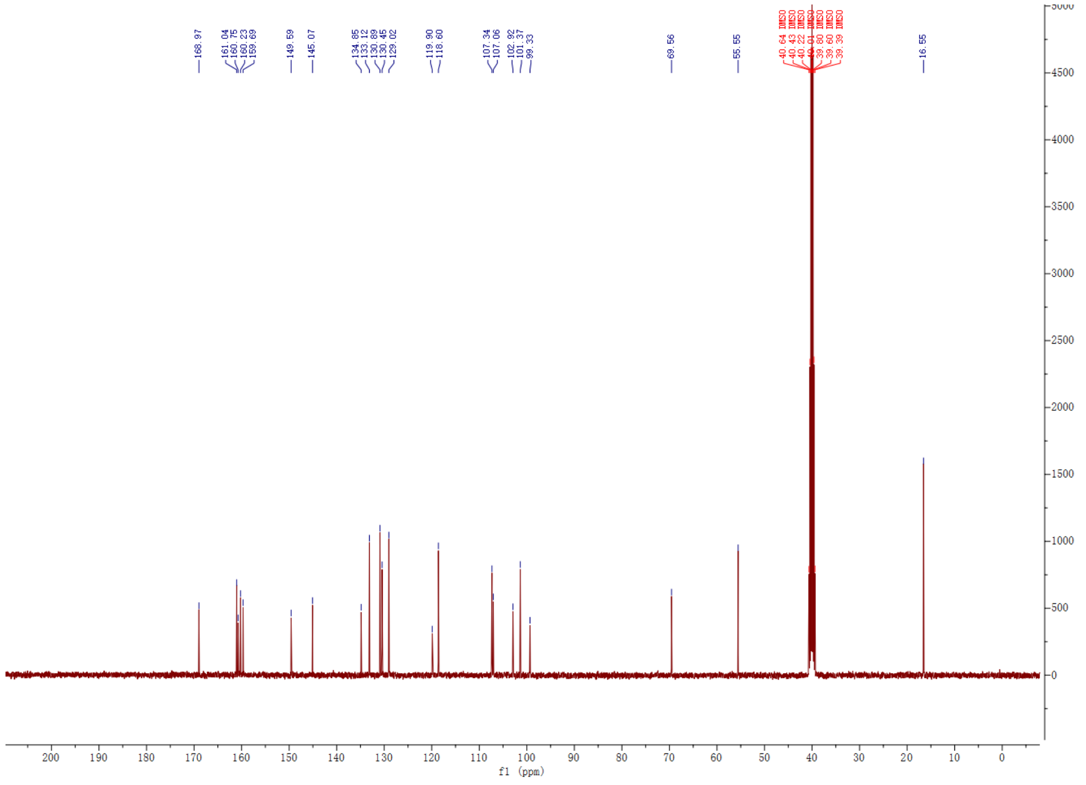

^{1}H and ^{13}C NMR spectra of TF8

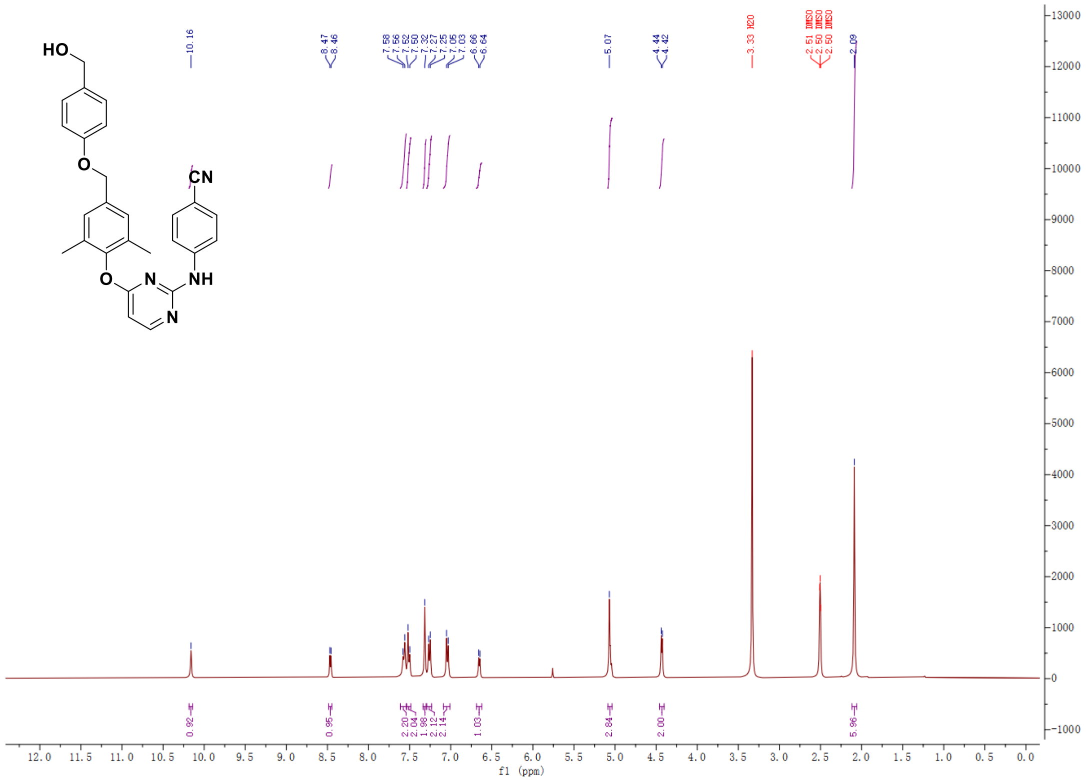

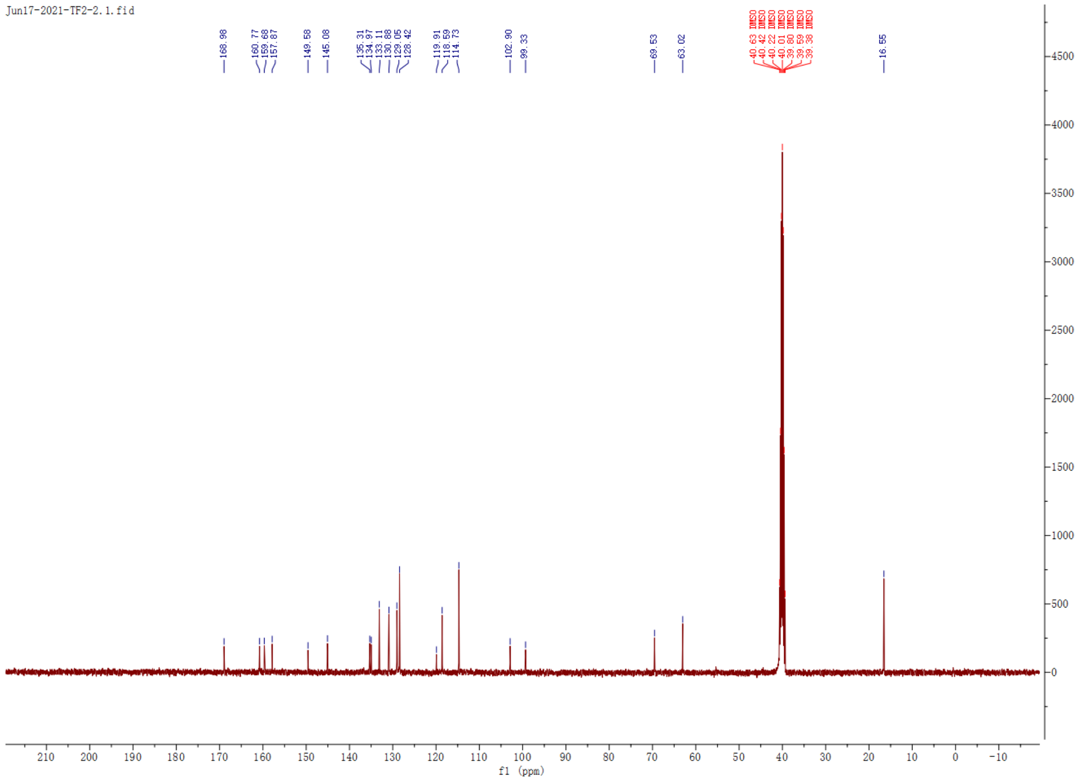

^{1}H and ^{13}C NMR spectra of TF9

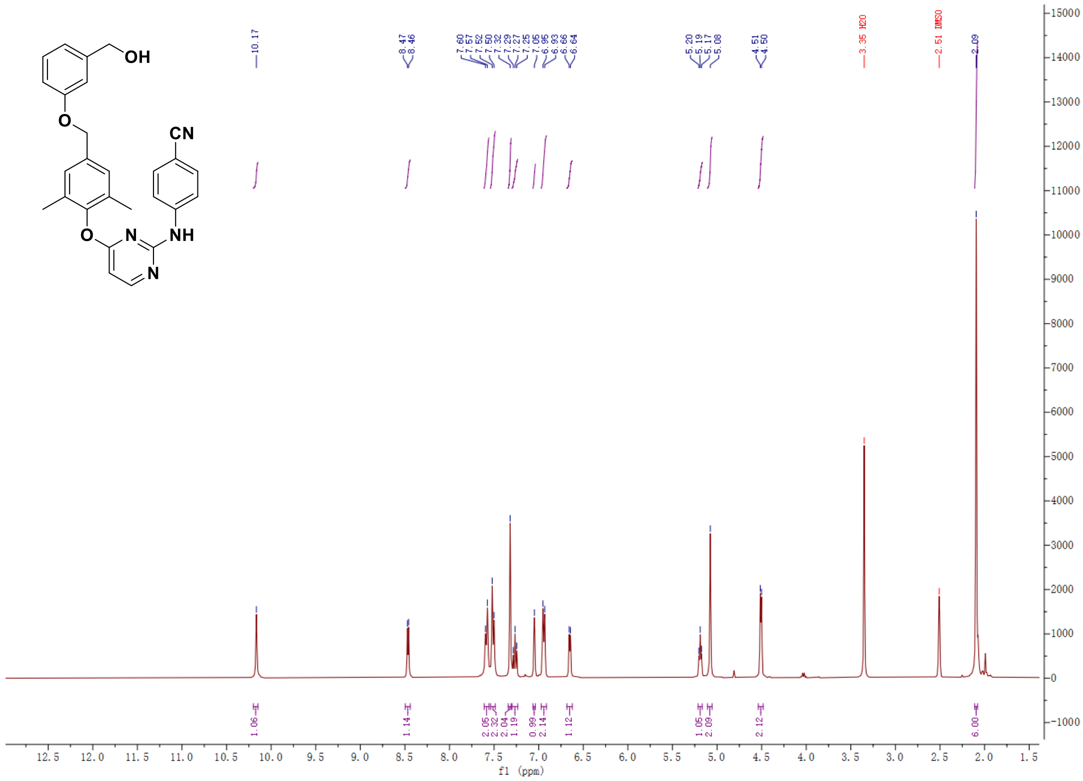

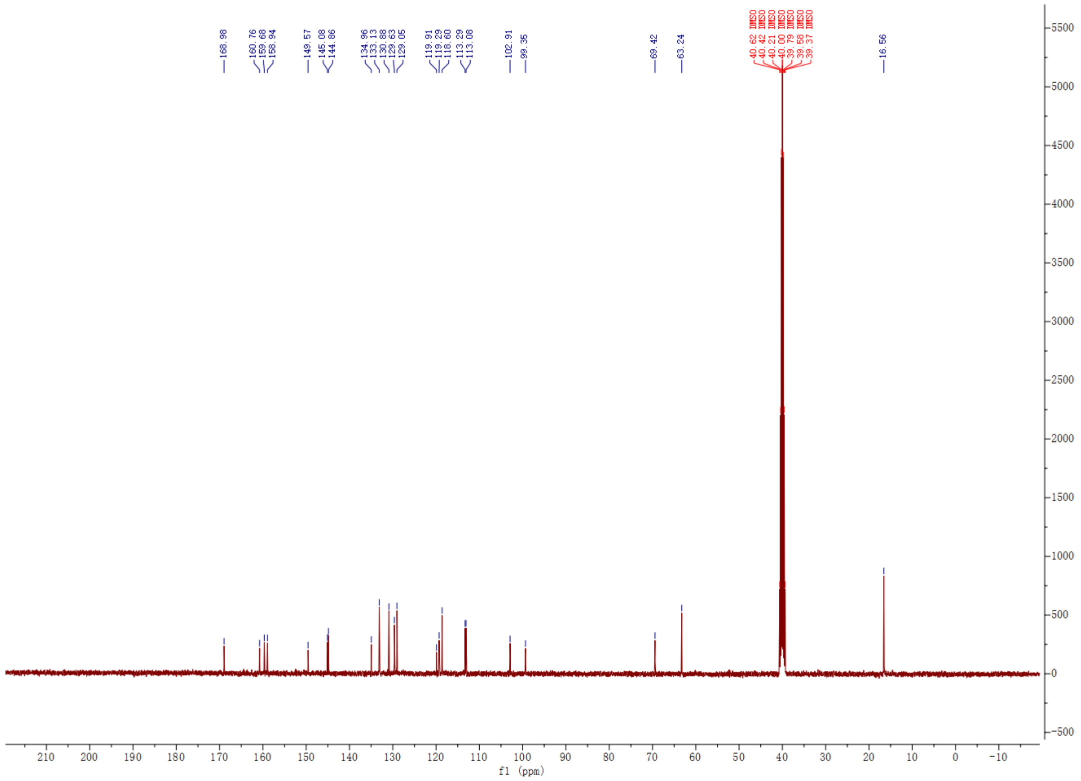

^{1}H and ^{13}C NMR spectra of TF10

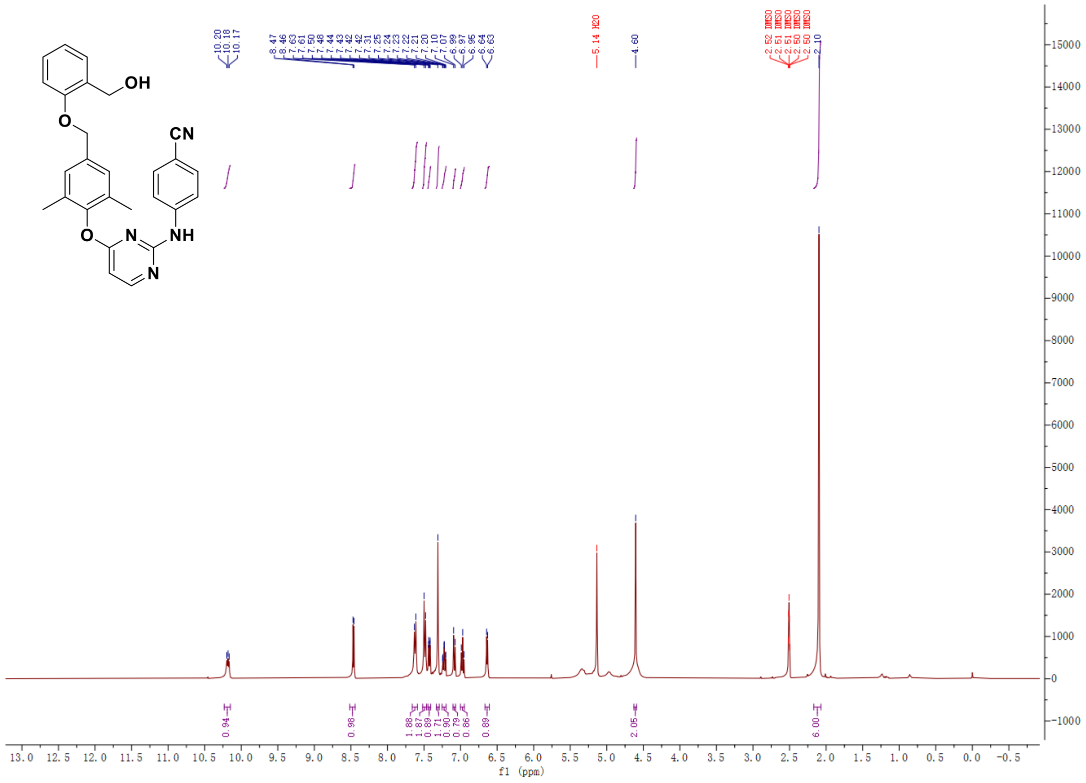

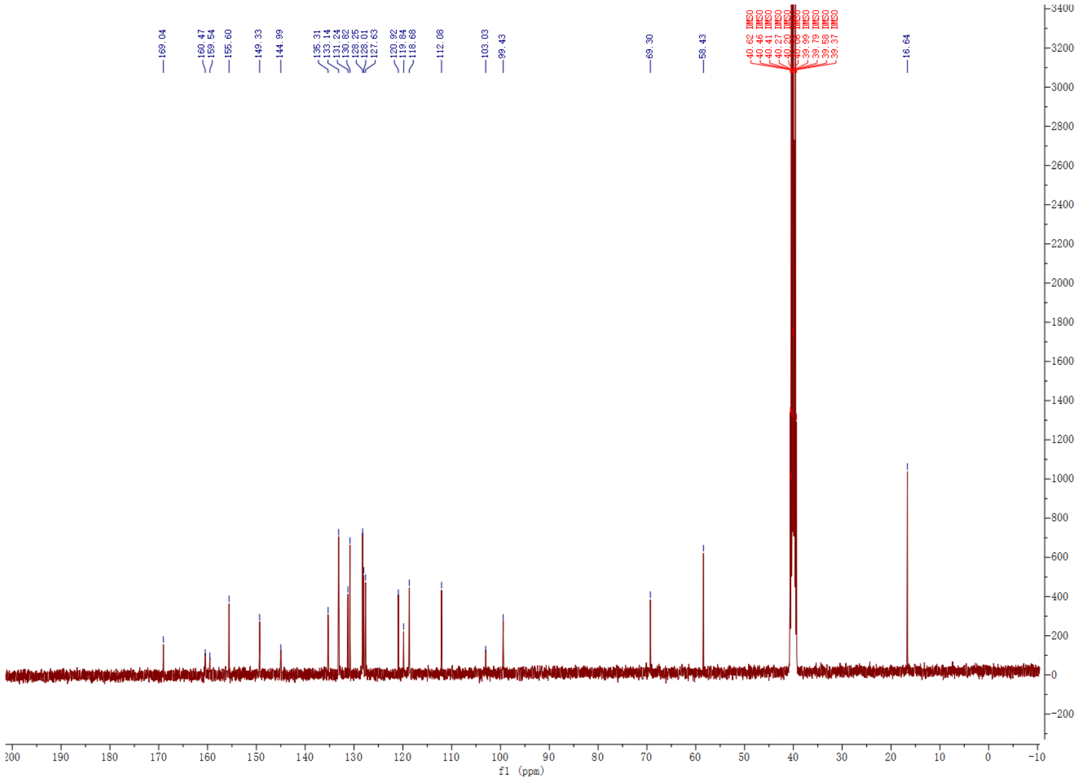

^{1}H and ^{13}C NMR spectra of TF11

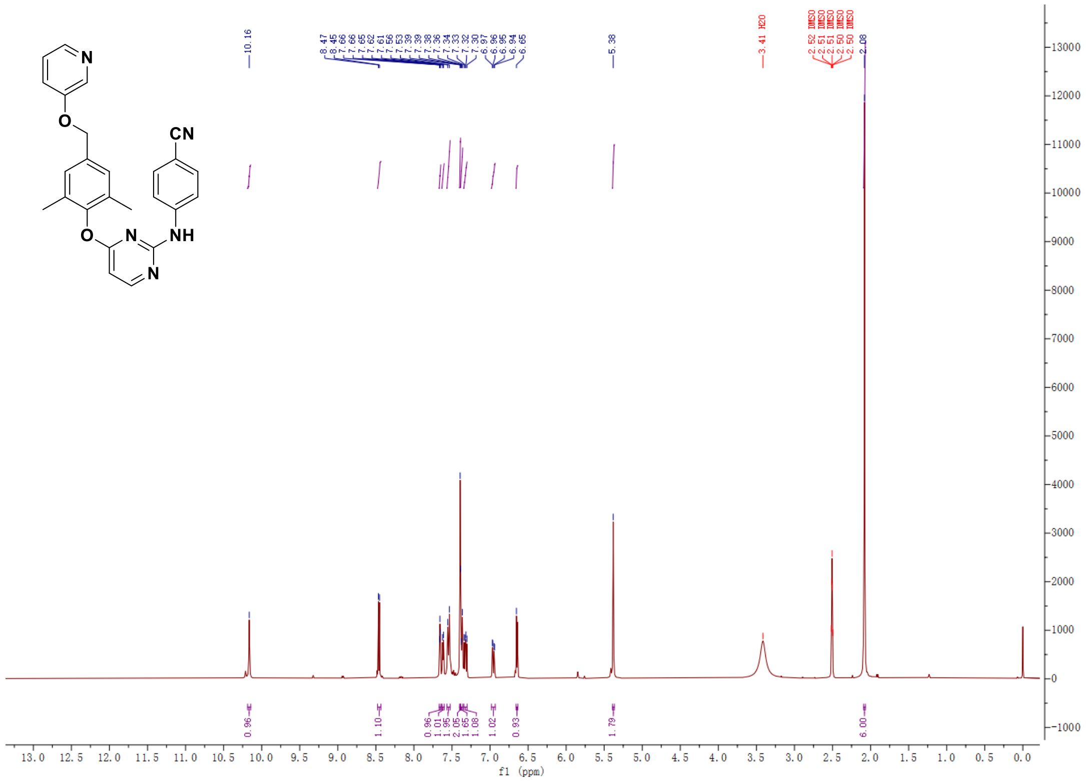

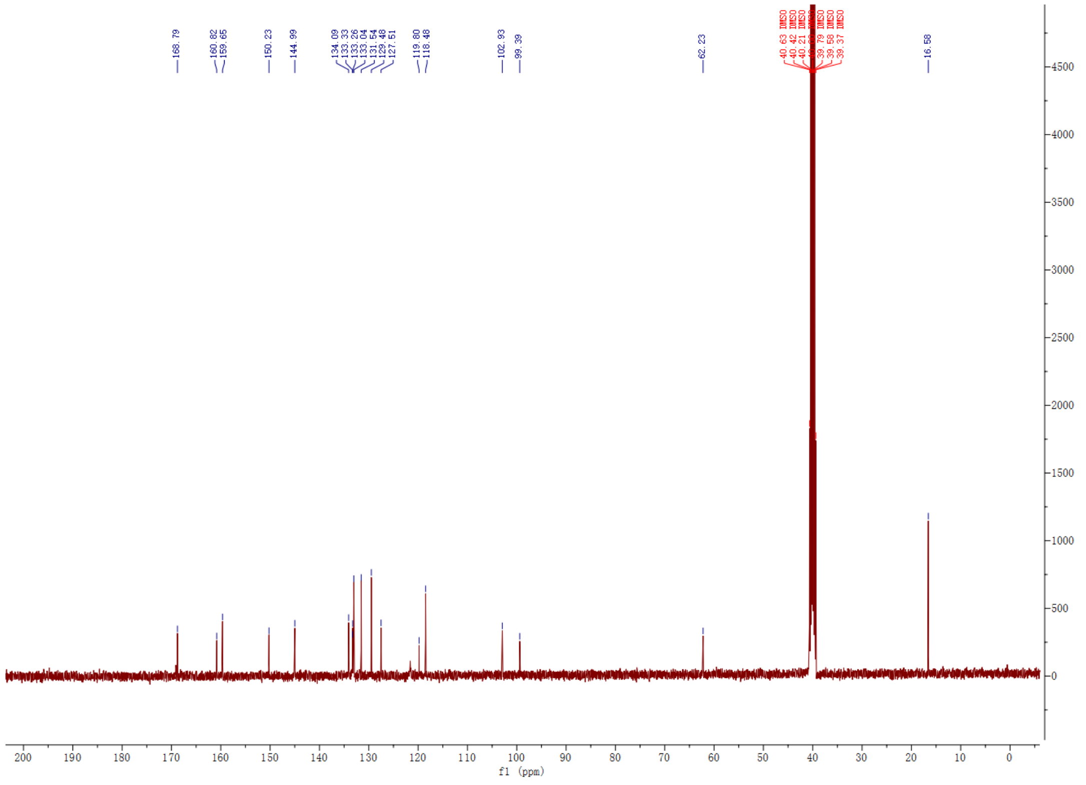

^{1}H and ^{13}C NMR spectra of TF12

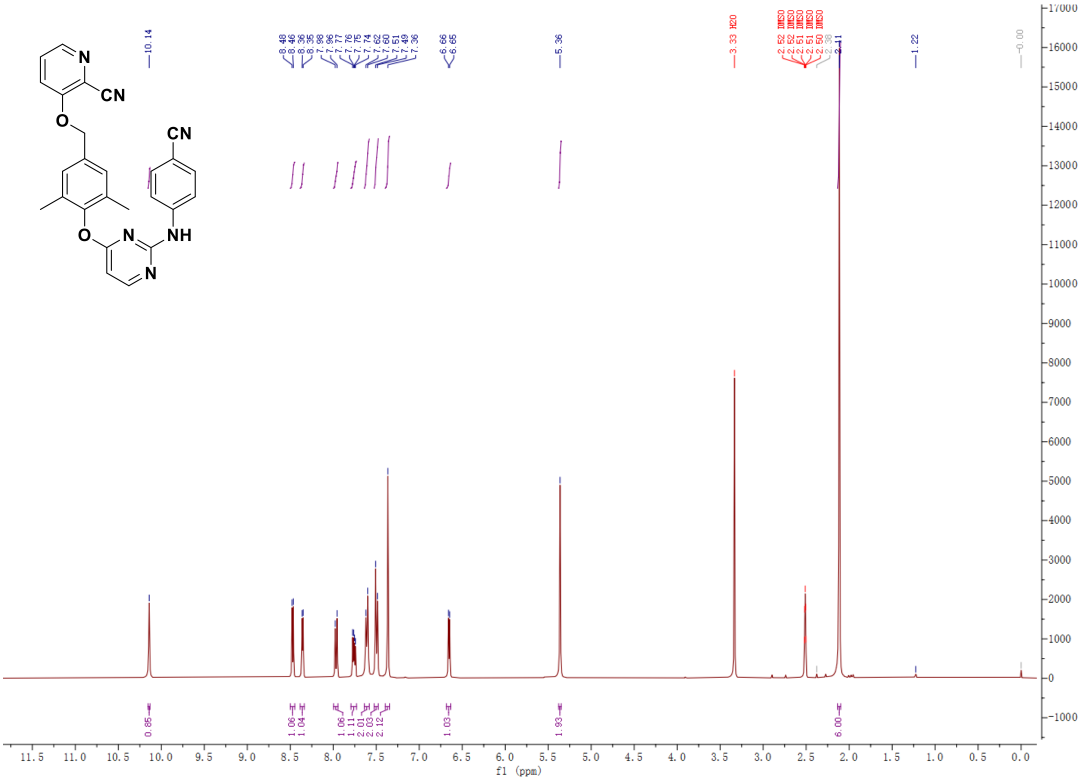

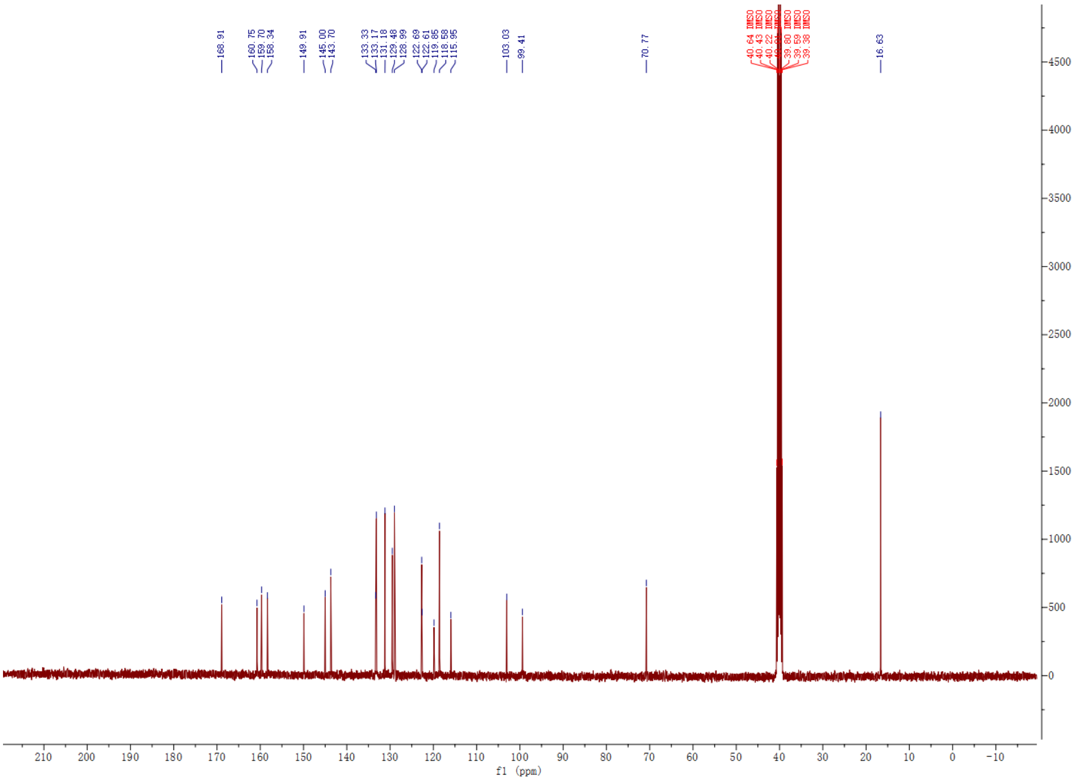

^{1}H and ^{13}C NMR spectra of TF13

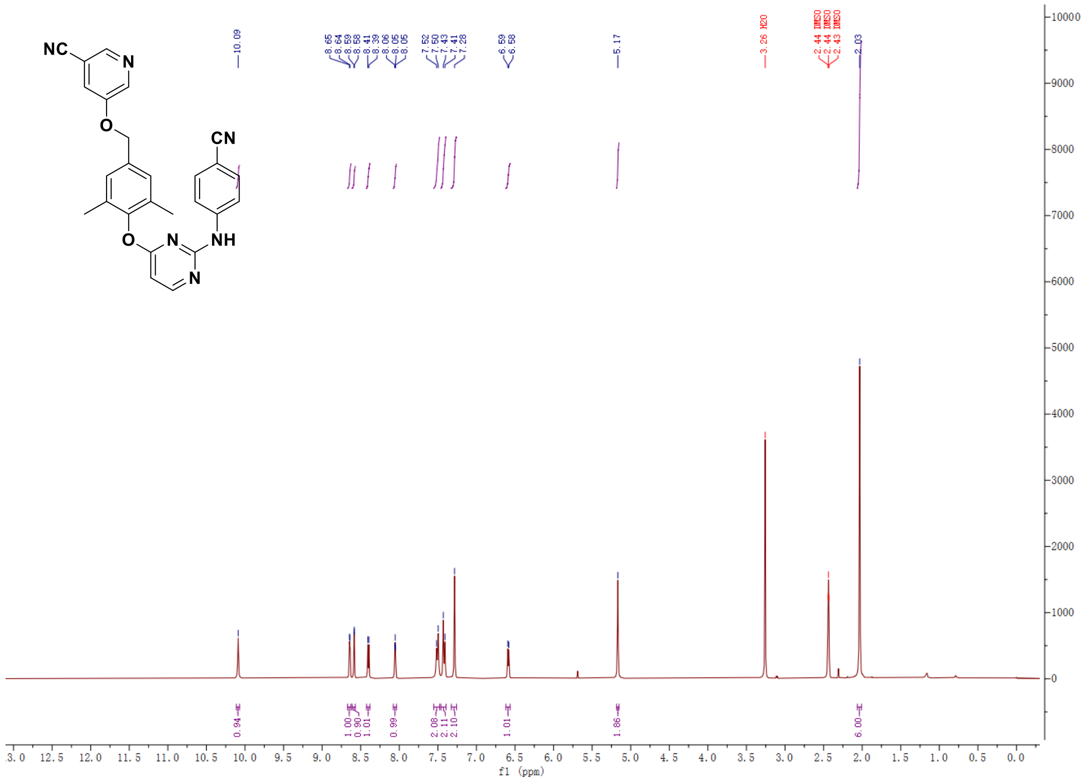

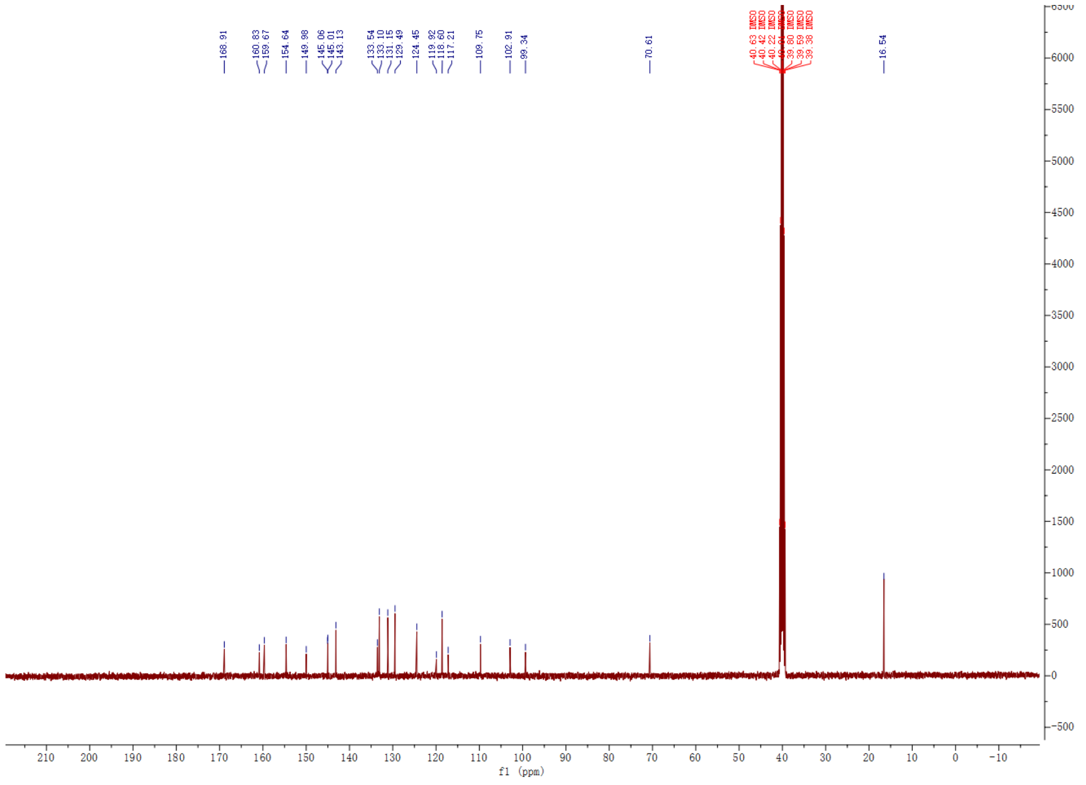

^{1}H and ^{13}C NMR spectra of TF14

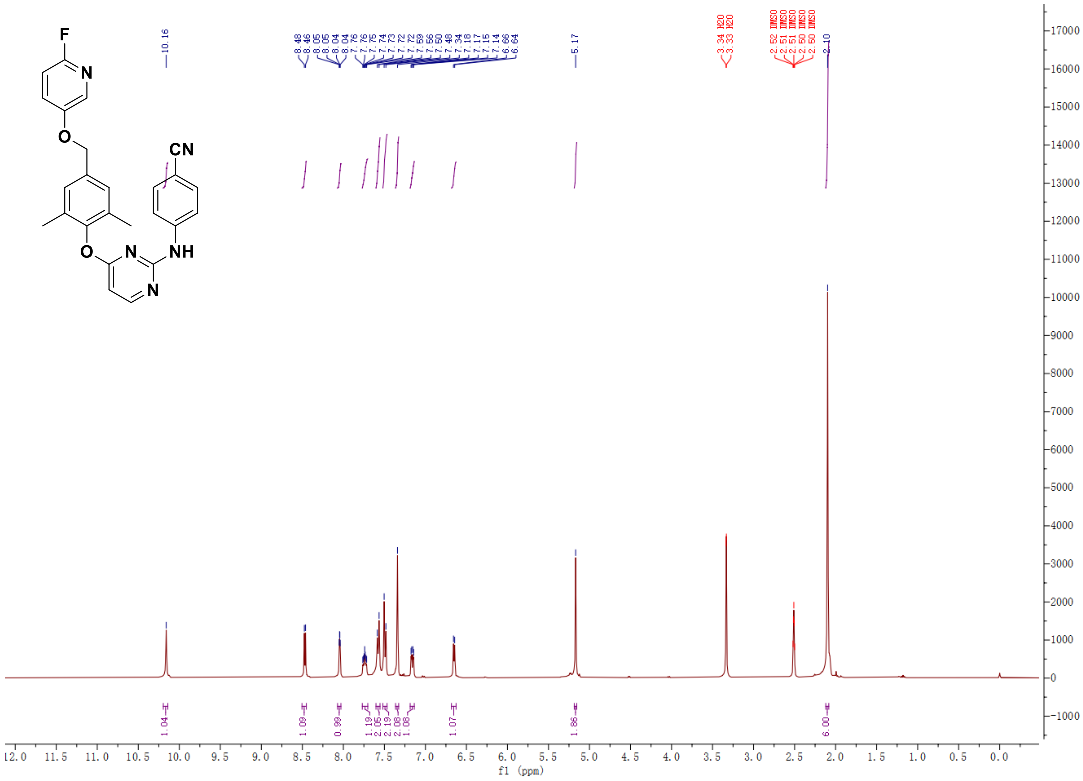

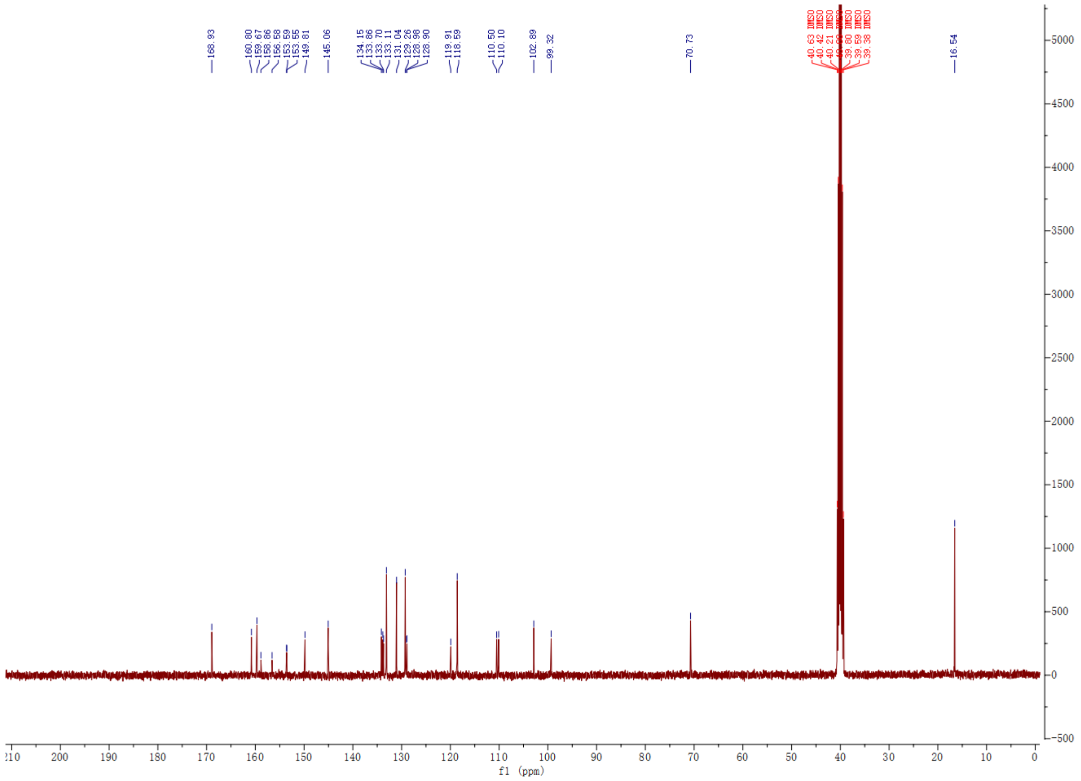

^1H and ^{13}C NMR spectra of TF15

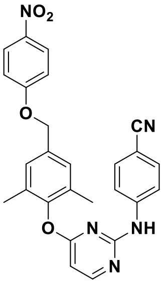

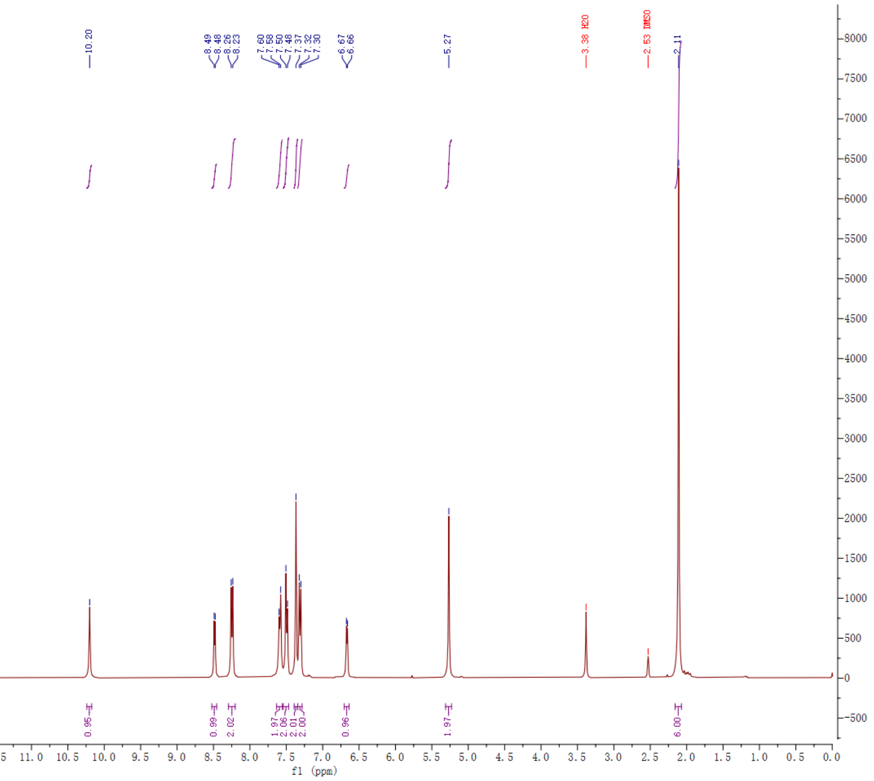

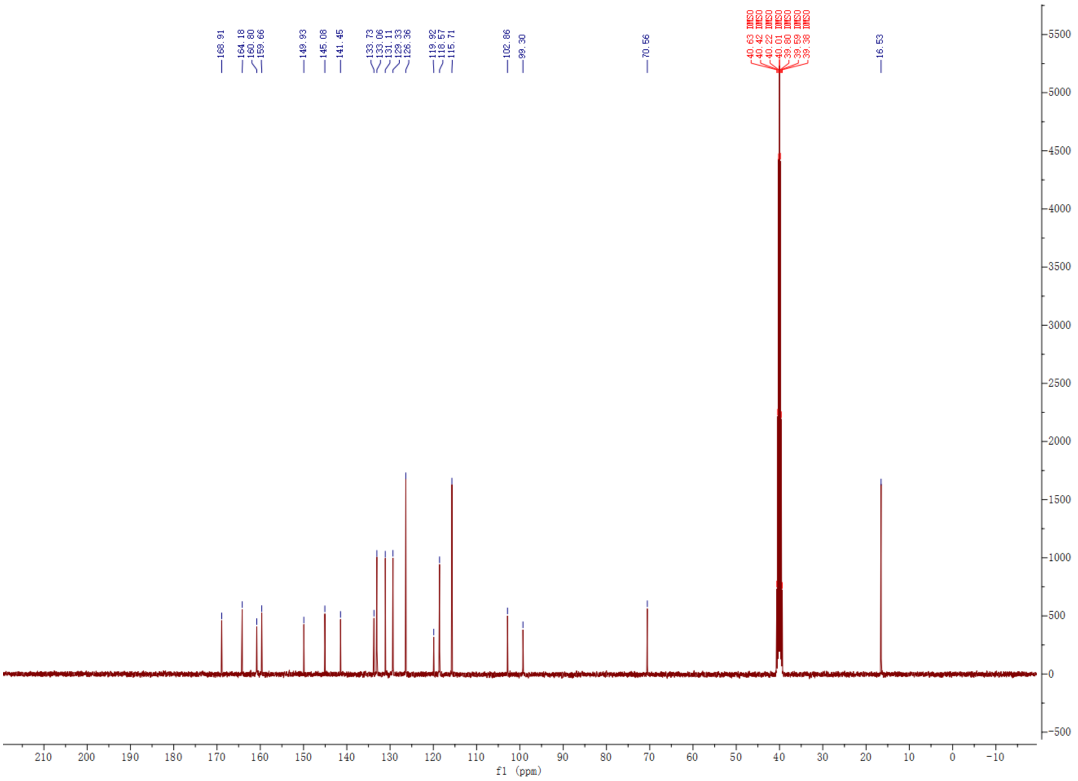

^{1}H and ^{13}C NMR spectra of TF16

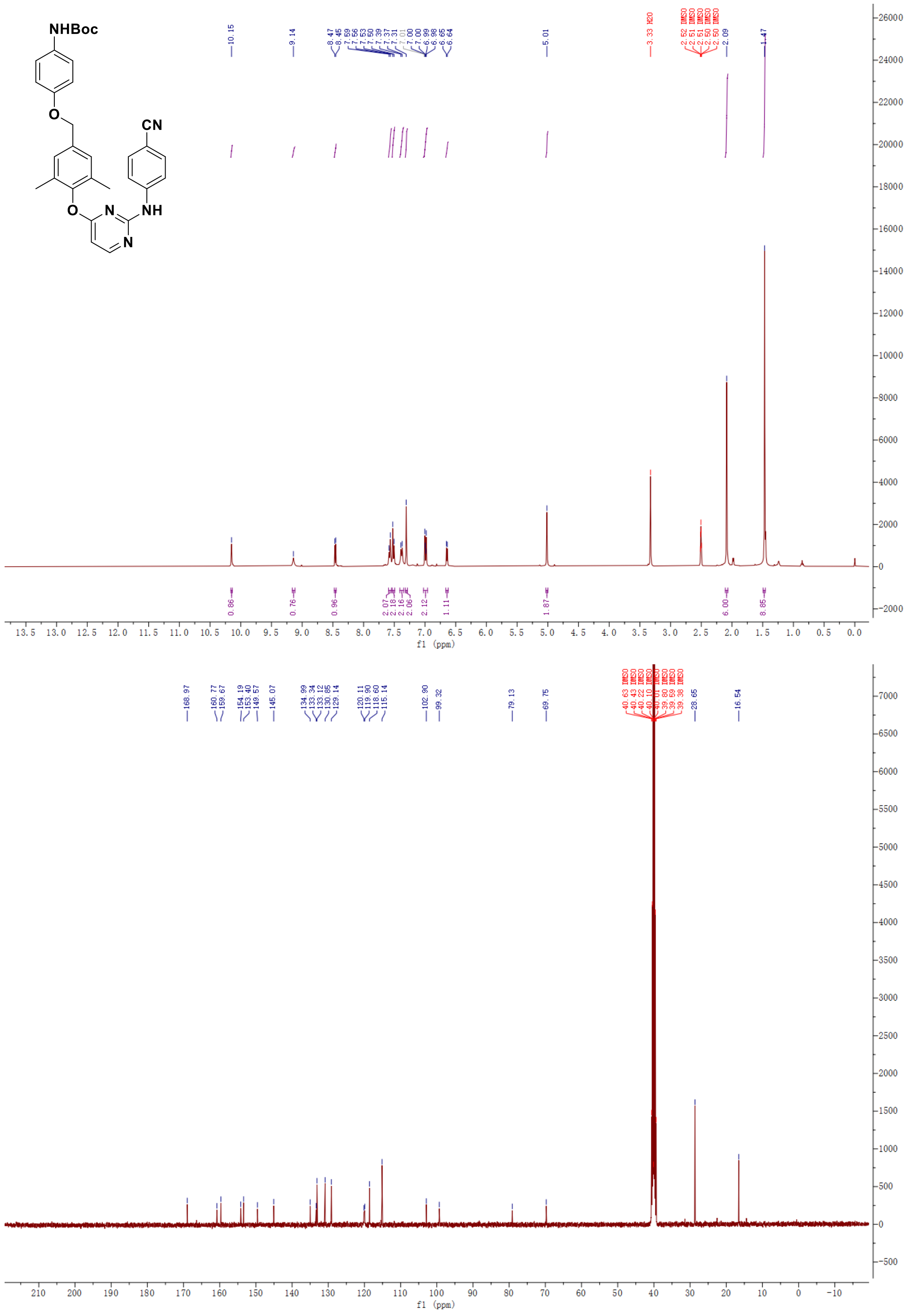

^{1}H and ^{13}C NMR spectra of TF17

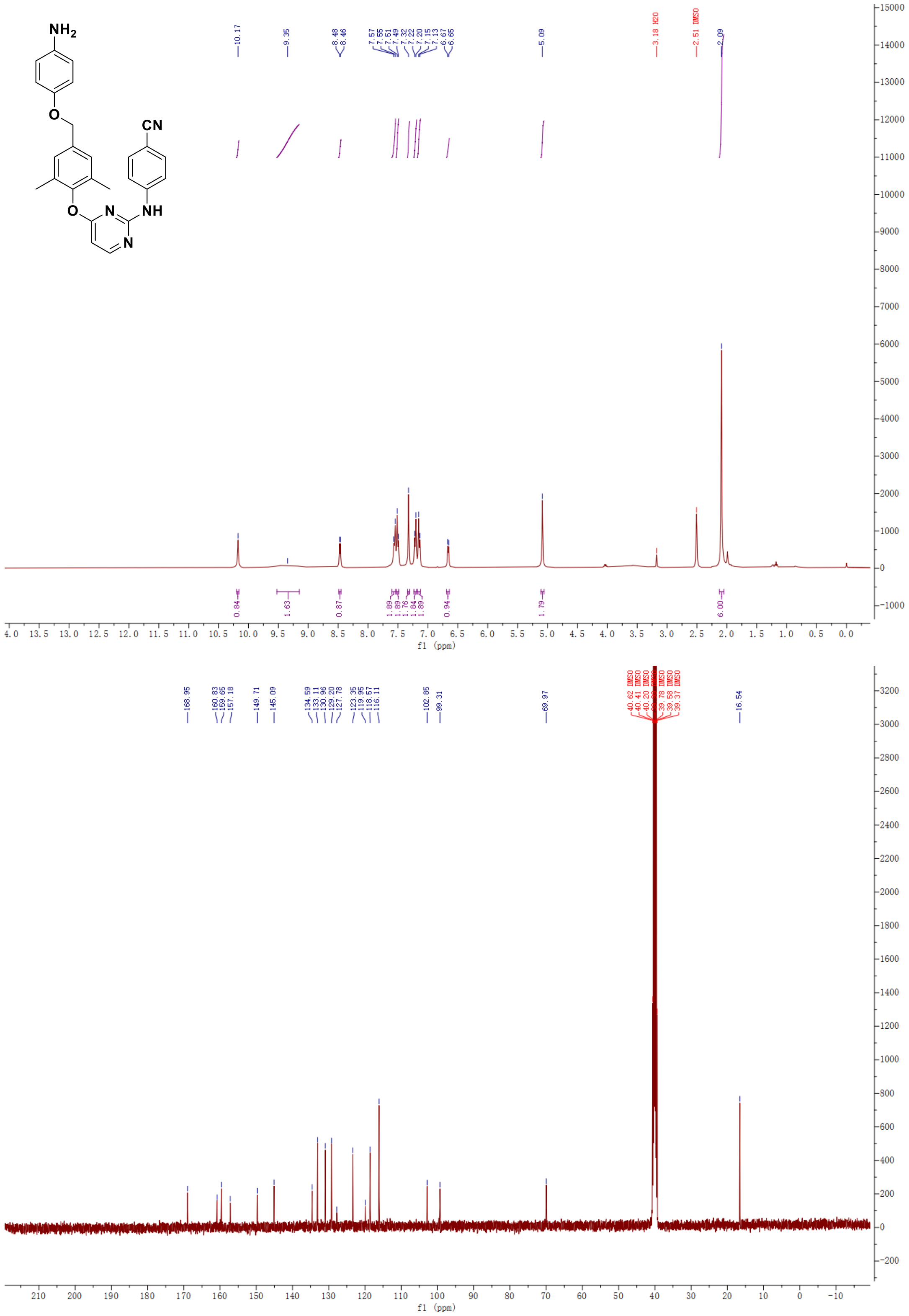

Supplement: Supplementary file 1 [file pharmaceuticals-15-01438-s001.zip › pharmaceuticals-1968260-supplementary.pdf]
